# Work from home and parenting: Examining the role of work‐family conflict and gender during the COVID‐19 pandemic

**DOI:** 10.1111/josi.12509

**Published:** 2022-06-26

**Authors:** Janine Bernhardt, Claudia Recksiedler, Anja Linberg

**Affiliations:** ^1^ Department of Social Monitoring & Methodology German Youth Institute Munich Germany

## Abstract

Many employers introduced or expanded working from home (WFH) in response to increasing infection rates after the onset of the COVID‐19 pandemic. Whether WFH enhances or depletes parents’ resources for their children is still an open question. Drawing on contextual models of parenting and demands‐resources approaches, we examine how WFH during the early stages of the COVID‐19 pandemic was linked to changes in responsive and harsh parenting, particularly in light of pandemic‐related increases in work‐to‐family conflicts (WFC). We further investigate gender differences in these associations. Our analyses draw on a sample of working parents from a large‐scale German family survey conducted in 2019 and a COVID‐19 follow‐up from 2020. Results from first difference regression models in combination with Heckman's sample selection method revealed strongly gendered patterns of changes in parenting. Specifically, responsive parenting decreased and harsh parenting increased only among mothers who did not work from home. In addition, WFH buffered increased spillovers from WFC on declines in responsive parenting among mothers. In contrast, fathers’ parenting remained largely unaffected by pandemic‐related changes in their work situation. We conclude that WFH can be a resource gain because it seems to have relieved some pandemic‐related parenting strain for mothers. Yet as a consequence, it may have reinforced gendered patterns of childcare. We discuss implications for policymakers and support services for families. We also place a special emphasis on those who are not able to work from home because this seems to have increased the risk that high work demands impaired their parenting during the early stages of the pandemic.

## INTRODUCTION

The onset of the COVID‐19 pandemic, along with the implementation of respective social distancing measures to contain its spread in many countries, have altered parents’ routines both in the family and work sphere fundamentally (Prime et al., 2020; Settersten et al., 2020). Many parents became fully and solely responsible for supervising, caring, and schooling their children—often during their regular working hours—due to the closure of childcare centers or schools and the ban to meet others outside of their household (e.g., grandparents or befriended families). A considerable share of employers further introduced or expanded working from home (WFH) in response to stay‐at‐home orders (Arntz et al., 2020). These arrangements allowed working parents to remain gainfully employed while compensating for the lack of formal childcare and closed schools. At the same time, these unprecedented conditions in work and family life posed new challenges to caregivers for engaging in supportive parent‐child interactions (Adisa et al., 2021; Chung et al., 2020; Goldberg, McCormick et al., 2021; Otonkorpi‐Lehtoranta et al., 2021; Schmeer et al., 2021).

Parenting is comprised of specific behaviors and emotional responses parents use in interactions with their children (Darling & Steinberg, 1993), which have long‐lasting consequences for the well‐being and development of children (e.g., Crouter & Bumpus, 2001; Nomaguchi & Milkie, 2020). The implications of WFH for parenting have not yet been studied, and particularly not during critical times such as the COVID‐19 pandemic. Recent evidence suggests that formal WFH options can free up time and psychological resources for parent‐child interactions (Kim, 2020; Kim et al., 2020). At the same, WFH can blur the boundaries between work and caregiving roles (Desrochers & Sargent, 2004). Managing these boundaries can, in turn, deplete parents’ resources with negative consequences for their parenting (Voydanoff, 2005a). WFH may therefore represent a resource gain or drain for parenting, which may further buffer or amplify the negative spillover of work‐related stress into parent‐child interactions. High levels of work‐to‐family conflicts (WFC) often have adverse effects on the health and well‐being of parents and can, in turn, lead to less responsive and harsher parenting (Borgmann et al., 2019; Chung et al., 2020; Haines et al., 2020; Hess & Pollmann‐Schult, 2020). However, whether WFH is effective in easing parents’ exposure to WFC or whether WFH instead intensifies the demands on parents even further is still an unresolved question. Lastly, because work and care roles are still highly gendered (Schoppe‐Sullivan & Fagan, 2020), WFH may affect mothers’ parenting more strongly than fathers’, particularly in situations with high work demands.

To fill these gaps, our study investigates how WFH during the early stages of the pandemic was associated with changes in parenting among mothers and fathers. Our theoretical framework is informed by contextual models of parenting, demands‐resources approaches, and role‐conflict theory, which all stress the interrelatedness of different life domains, such as work and family (Belsky, 1984; Belsky & Jaffee, 2006; Bronfenbrenner, 1999; Bronfenbrenner & Morris, 2006; Demerouti et al., 2001; Greenhaus & Beutell, 1985; Voydanoff, 2005a). Based on previous research that conceptualized WFH to be either a demand or a resource (Kim et al., 2020; Voydanoff, 2005a), we test competing hypotheses on the joint impact of WFH and WFC on parenting. We further examine whether and how these work conditions (i.e., the use of WFH arrangements and the level of WFC) interact with gender to contribute to differential changes in parenting. Our study draws on large‐scale German survey data collected right before and during the pandemic. Gender differences in the role of WFH and its interplay with WFC on parenting may be particularly pronounced in countries like Germany where welfare state policies and cultural beliefs still support gendered work‐care arrangements (Grunow et al., 2018).

## THEORETICAL CONSIDERATIONS ON PARENTING

This study investigates responsive and harsh parenting as two key dimensions of the socioemotional quality of parent‐child interactions (Grusec, 2011). Parenting is comprised of specific behaviors and emotional responses parents use in interactions with their children (Darling & Steinberg, 1993) and is one of the most influential forces that shape child development and adjustment (Buehler, 2020; Nomaguchi & Milkie, 2020). There is a long tradition of research that groups different types of parental behaviors and responses into distinct dimensions (Kuppens & Ceulemans, 2019) or classifies them into typologies (Baumrind, 1991). These dimensions range from responsive parenting, indicated by being supportive, involved, emotionally available for the child, and using child‐centered communication, to harsh parenting consisting of disciplinary strategies, such as verbal or physical punishments (Kuppens & Ceulemans, 2019; Maccoby & Martin, 1983). Associations between these dimensions and child outcomes vary starkly. For example, studies found that warm and supportive parenting was linked to higher levels of children's academic success and lower levels of behavioral problems (Amato & Fowler, 2002; Spera, 2005), whereas harsh parenting was associated with higher levels of child aggression and externalizing problems (Buehler, 2020; Hess & Pollmann‐Schult, 2020).

However, parenting as a process occurring in the familial context cannot be viewed in isolation from various external factors. While a large number of studies examined intra‐familial characteristics, contextual models also point to the importance of other life domains and environments (e.g., Lerner et al., 2002). For example, Bronfenbrenner and Morris (2006) emphasized not only the importance of parenting itself but also the embeddedness of parenting in contexts. Parenting is viewed as a proximal process in terms of reciprocal interactions between one person and their immediate external (personal) environment or context. Contexts are viewed as systems nested within and interrelated with each other, which highlights that patterns of interactions within these systems influence each other. Following this idea, different models, such as the *process model of parenting*, argue that parenting is not only shaped by characteristics of the involved persons (i.e., parent and child) in the family sphere, but also by broader social contexts (e.g., the occupational experiences of parents; Belsky, 1984; Belsky & Jaffee, 2006). This means that experiences of support or stress in one domain (e.g., work) can affect parents’ behaviors in another domain (e.g., parenting).

### WFH as a resource gain or drain for parenting during the pandemic

In response to increasing infection rates after the onset of the COVID‐19 pandemic, many employers introduced or expanded WFH arrangements. The share of employees working from home increased noticeably during stay‐at‐home orders, which required people to leave the house only if they were unable to work from home and for other important reasons (Arntz et al., 2020; Brynjolfsson et al., 2020). WFH was not only implemented as a social distancing measure, but also to allow working parents to reorganize work and care duties, and to remain gainfully employed while compensating for reduced childcare and closed schools. Switching to WFH during the pandemic increased the interactions between family members considerably (Kramer & Kramer, 2021) and was likely to affect parents’ emotional behaviors towards their children as well. Evidence on the implications of WFH for parenting is still lacking. However, WFH has been one of the most debated ways out of the work‐family‐balance dilemma for about two decades (Chung & van der Lippe, 2020; Perry‐Jenkins & Gerstel, 2020).

Work‐family research is still inconclusive on whether WFH represents a resource gain or drain for parents. On the one hand, WFH has been conceptualized as a flexible work arrangement that can increase parents’ control over where and/or when they work (Allen et al., 2014) and can enable them to reorganize work and care responsibilities more freely (Kossek et al., 2006). Studies showed, for example, that WFH was associated with less commute time, increased autonomy, better work‐family balance, and a more frequent engagement of parents in enrichment activities with their children (Allen et al., 2013; Arntz et al., 2019; Hill et al., 2003; Kim, 2020; Kim et al., 2020; Kossek et al., 2006; Schieman & Young, 2010). On the other hand, WFH has been theorized as a boundary‐spanning demand (Voydanoff, 2005a, 2005b), which requires parents to actively manage spatial and temporal boundaries between work and other life domains (Kossek et al., 2012). These boundaries tend to become more permeable when parents work from home (Desrochers & Sargent, 2004), which can also lead to increased multitasking, longer working hours, WFC, and stress, particularly if WFH was used for supplemental work done during overtime hours (Abendroth & Reimann, 2018; Arntz et al., 2019; Kim et al., 2020; Ojala et al., 2014; Schieman & Young, 2010; Song & Gao, 2020).

Drawing on this pre‐pandemic research, WFH could have either eased or amplified the spillover of pandemic‐related stress on parenting caused by social distancing measures, novel daily hassles, and health‐related fears (Cusinato et al., 2020; Settersten et al., 2020; Vaterlaus et al., 2021). On the positive side, many employers started establishing WFH as the new normal for those who were able to perform their jobs offsite (Kramer & Kramer, 2020), which likely led to an expansion of formal WFH arrangements as part of one's regular working hours. In contrast to WFH for supplemental work outside of regular working hours (Chung & van der Lippe, 2020; Fenner & Renn, 2010), formal WFH arrangements supported by organizational leaders can offer parents greater flexibility and security in their work‐family management (Hill et al., 2003; Kelly et al., 2008; Kim et al., 2020). Formal WFH options can therefore free up time and psychological resources for parent‐child interactions (Kim, 2020). Qualitative studies reported that one theme in parents’ experiences during early stages of the pandemic revolved around time savings and increased flexibility through WFH, which helped them spend more time, connect, and bond with their children (Adisa et al., 2021; Lantsoght et al., 2021; Salin et al., 2020; Sánchez‐Mira et al., 2021). Studies further identified parent‐child communication as an important coping strategy to help children understand the lockdown measures and to cope with their emotions such as anxiety and frustration (Salin et al., 2020; Vaterlaus et al., 2021). From this perspective, WFH may represent a resource that buffered against pandemic‐related decreases in responsive parenting and increases in harsh parenting.

On the downside, however, managing spatial and temporal boundaries between work and caregiving roles became particularly challenging for parents who switched to WFH during the pandemic (Cusinato et al., 2020; Otonkorpi‐Lehtoranta et al., 2021; Prime et al., 2020; Sánchez‐Mira et al., 2021; Settersten et al., 2020). Qualitative research found that these parents started working in sequential shifts and during atypical times, such as during early mornings, evenings, and even nights to manage childcare and homeschooling during the daytime (Lantsoght et al., 2021; Salin et al., 2020). Quantitative studies linked this dual burden to increased levels of stress and lower psychological well‐being among parents (Goldberg, McCormick et al., 2021; Huebener et al., 2021) and, in turn, to more negative and harsher parenting (Chung et al., 2020; Vaterlaus et al., 2021). From this perspective, WFH represented an additional stressor, which depleted parents’ resources and reinforced pandemic‐related declines in parenting.

Because research on work‐family reconciliation and parent‐child relationships provides reasonable arguments in both directions—WFH as a resource gain or drain—we propose two competing hypotheses:

Hypothesis 1a.: WFH represents a *resource gain* for parents and is therefore positively associated with responsive parenting and negatively associated with harsh parenting.
Hypothesis 1b.: WFH represents a *resource drain* for parents and is therefore negatively associated with responsive parenting and positively associated with harsh parenting.

### WFH and parenting in the context of WFC

Because most parents continue to be actively engaged in gainful employment after some period of parental leave after childbirth in many Western nations (Perry‐Jenkins & Gerstel, 2020), balancing the demands that stem from both the family and work sphere remains a key challenge for parents. Conflicts resulting from interfering demands are understood as contextual stressors, which have also been linked to parenting (Crouter & Bumpus, 2001; Nomaguchi & Milkie, 2020). Role‐conflict theory describes WFC as a type of interrole conflict caused by work demands, such as time pressures, psychological strain, or role expectations, which compete with role expectations in the family (Carlson et al., 2000; Greenhaus & Beutell, 1985). The spillover hypothesis further proposes that when work demands interfere with family responsibilities, the resulting role conflicts deplete parents’ time and emotional resources, produce strain, and impair their functioning in the family sphere by undermining parents’ mental or emotional availability for their children (Bakker & Demerouti, 2013; Matias et al., 2017; Voydanoff, 2005a).

The COVID‐19 pandemic has altered the experience of WFC for many parents (Verweij et al., 2021). The economic and organizational consequences of the pandemic likely increased work demands for parts of the workforce irrespective of whether they worked in or outside of the home (e.g., workers in healthcare or teaching occupations), and decreased work demands for others, particularly those in work organizations that were temporarily closed (e.g., Arntz et al., 2020; Fana et al., 2020). Studies before and during the pandemic showed that WFC was indeed related to higher levels of stress (Freisthler et al., 2021) and health problems (Borgmann et al., 2019). Although some research did not find that WFC had negative spillover effects on parenting (van den Eynde et al., 2020; Verweij et al., 2021), several studies found that higher levels of WFC were associated with less responsive and harsher parenting (Cooklin et al., 2015; Haines et al., 2020; Hess & Pollmann‐Schult, 2020). In line with this evidence for the spillover hypothesis, we expect the following:

Hypothesis 2.: WFC is negatively associated with responsive parenting and positively associated with harsh parenting.

Furthermore, WFC and WFH may jointly shape parenting in two major ways. First, if WFH represents a resource gain for parents as proposed in Hypothesis 1a, this work arrangement may be particularly relevant in the context of high exposure to WFC. WFH may then ease parents’ exposure to high levels of WFC by allowing them more control and flexibility in handling work and care demands (Hill et al., 2003; Kim et al., 2020). This argument is supported by the job‐demands‐resources model (Bakker & Demerouti, 2007; Demerouti et al., 2001), which postulates that the spillover of WFC into family life can be reduced by job resources buffering the negative effects of high work demands. In turn, the protective effect of resources neutralizes the negative impact of demands in a way that “demands no longer contribute to outcomes” (Voydanoff, 2008, p. 46). From this perspective, WFH may enable parents to respond to their children's needs immediately, even in situations with a high load of work demands. As a result, parents’ interactions with their children may be less susceptible to fluctuations in WFC when they work from home rather than onsite.

Second, if WFH represents a resource drain as proposed in the competing Hypothesis 1b, this work arrangement may be particularly harmful to parent‐child interactions in the context of high levels of WFC. This argument has emerged specifically in research on parenting during the pandemic, which suggested that parents who experienced high work demands may have been less successful in preventing spillover of WFC because they were largely bound to the home and had fewer opportunities for recreation and social contacts as important coping strategies (Verweij et al., 2021). Studies conducted during the pandemic further reported that parents found respite when they were able to spend some time away from the home and to have a moment to themselves (Freisthler et al., 2021; Salin et al., 2020). More generally, WFH coupled with high work demands may blur work‐family boundaries even further, reduce parents’ resources for their children and, in turn, diminish their responsiveness and trigger harsher parenting (Prime et al., 2020). Thus, the resource drain for parenting may become even stronger with increasing levels of WFC when parents work from home rather than onsite.

Parallel to the two competing arguments theorizing WFH as either a resource gain or drain, we formulate the two following hypotheses:

Hypothesis 3a.: The negative relationship between WFC and responsive parenting, as well as the positive relationship between WFC and harsh parenting, is attenuated when parents work from home rather than onsite.
Hypothesis 3b.: The negative relationship between WFC and responsive parenting, as well as the positive relationship between WFC and harsh parenting, is amplified when parents work from home rather than onsite.

### Gender differences in the impact of WFH and WFC on parenting

Gender research during the pandemic has been largely concerned with the aggravation of gender inequality in many Western nations. Before the onset of the COVID‐19 pandemic, mothers, on average, already shouldered a larger part of care work than fathers despite overall trends of increasing maternal labor market participation and paternal involvement in childcare (Perry‐Jenkins & Gerstel, 2020; Schoppe‐Sullivan & Fagan, 2020). The pandemic may have ruptured these trends. Several studies reported persisting or even increased gender gaps in employment and childcare. For example, studies from Germany, Italy, the UK, and the U.S. showed that mothers worked on average fewer hours in paid employment and spent more hours on childcare, homeschooling, and household chores compared to fathers during strict lockdown periods (see, e.g., Collins et al., 2021; Del Boca et al., 2020; Hank & Steinbach, 2021; Hipp & Bünning, 2021; Kreyenfeld & Zinn, 2021; Petts et al., 2021; Sevilla & Smith, 2020). Although research has still largely focused on heterosexual two‐parent families, some studies suggest that increasing gender divides during the early stages of the pandemic also occurred in other family types. Studies reported that separated mothers faced intensified parenting demands resulting from difficulty coordinating and cooperating with their ex‐partners, particularly with regard to homeschooling (Goldberg, Allen et al., 2021). In addition, initial evidence suggests that even though same‐sex couples tended to report more fairness in their division of unpaid work before and during the pandemic, lesbian mothers reported higher concerns than heterosexual mothers and gay fathers that they were not contributing their fair share of care work (Craig & Churchill, 2021; Evertsson et al., 2021).

WFH seemed to play an important role in the development of the gender gap in care work and may therefore have gendered implications for parenting as well. Pre‐pandemic research suggested that instead of enabling parents to share care work more equally, WFH appeared to reinforce the gendered division of labor among heterosexual couples. Quantitative evidence showed that WFH on a regular basis was associated with spending more time on childcare and household chores for mothers only (Powell & Craig, 2015). Studies during the pandemic indicated only little changes in the gendered impact of WFH (Shockley et al., 2021) and showed that particularly mothers who worked from home maintained or extended their time spent on childcare. The impact of WFH on fathers’ involvement, however, was less pronounced and tended to depend on their partners’ work situation (Del Boca et al., 2020; Hank & Steinbach, 2021; Hipp & Bünning, 2021; Zoch et al., 2021). Pandemic‐related research on post‐separation families reported similar gendered patterns in the division of childcare and homeschooling among separated mothers who worked from home and their ex‐partners (Goldberg, Allen et al., 2021).

Persisting cultural norms of the male‐breadwinner/female‐caregiver model can be considered as a major driving force behind the gendered division of labor, which have also been used to explain the gendered impact of WFH. More specifically, the breadwinner‐caregiver model was the dominant work‐family model for decades (Lewis, 2001) and has still not lost its normative power. Despite general trends of expanding gender‐egalitarian attitudes and cultural differences, the breadwinner‐caregiver model is still highly salient, even among young people (e.g., Grunow et al., 2018; McConnon et al., 2021). This is partly because children adopt gender role behaviors from their parents as influential role models (e.g., Fulcher et al., 2015; Moen et al., 1997). From an intersectional perspective, power relations within and outside the family uphold by gendered institutional structures such as in law, politics, and the economy (Acker, 1992) reinforce gender differences in family roles (Few‐Demo & Allen, 2020). Through this lens, gender relations can further interact with race, ethnicity, class, family structure, or sexuality to produce distinct outcomes with regard to gender inequalities (Perry‐Jenkins & Gerstel, 2020). Gender norms at the societal level are also echoed by workplace cultures (Ferree, 2010). Organizations are often still oriented towards the male breadwinner as the *ideal worker*, who can prioritize work over family and therefore relies on a (female) caregiver organizing paid work around care demands (Acker, 1990; Chung & van der Lippe, 2020; Williams, 2000). Cultural gender norms are so deeply ingrained in society that their impact is not limited to biological two‐parent families, but has been documented for same‐sex couples and post‐separation families as well (Craig & Churchill, 2021; Evertsson et al., 2021; Goldberg, Allen et al., 2021).

Evidence on the gendered impact of WFH on parenting is still sparse. Consistent with findings on the gendered division of childcare, a pre‐pandemic study by Kim (2020) showed that mothers, but not fathers, engaged in enriching activities with their children more frequently if they used formal WFH arrangements offered by their employers. Qualitative studies further demonstrated that parents’ strategies for using WFH options can differ by gender. Mothers may work from home predominantly to facilitate work‐family reconciliation, whereas fathers may work from home primarily to increase their work performance (Sullivan & Lewis, 2001; Sullivan & Smithson, 2007). This is in line with research findings during the pandemic demonstrating that particularly women across different family forms (e.g., different‐sex and same‐sex couples, single‐parent families) used work from home as a strategy for managing work and care demands when childcare institutions and schools were closed during lockdowns (Craig & Churchill, 2021; Goldberg, Allen et al., 2021; Goldberg, McCormick et al., 2021).

Against this backdrop, we expect mothers’ parenting to be more affected by WFH than fathers’ parenting. Furthermore, irrespective of whether WFH represents a resource or demand in the context of increased levels of WFC, we anticipate that this effect will be stronger among mothers than fathers. Findings during the pandemic highlighted both positive and negative effects of increased work and care demands for mothers who worked from home (Craig & Churchill, 2021; Goldberg, McCormick et al., 2021). The increased role overlap concerning work and family diminished their performance, yet simultaneously enhanced the quality of their family relationships (Adisa et al., 2021). In addition, pre‐pandemic evidence found stronger feelings of parenting guilt in the context of high levels of WFC among mothers only (Borelli et al., 2017). Due to the stronger overlap of work and care roles among mothers compared to fathers, we expect gender differences to be particularly pronounced among parents who experienced increased levels of WFC.

Hypothesis 4.: The relationship between WFH and parenting is stronger among mothers compared to fathers.
Hypothesis 5.: The impact of the interplay between WFH and WFC on parenting is more pronounced among mothers compared to fathers.

## STUDY CONTEXT

The institutional and cultural context of this study could affect its results. Our study is set in Germany, which is characterized by welfare state institutions, family policies, and gender beliefs that promote rather traditional work‐care arrangements among parents (Grunow et al., 2018). For instance, taxation and social security systems leave little fiscal benefits for dual‐earner couples (Lechevalier, 2019) and instead provide incentives for the one‐and‐a‐half‐earner model (Lewis, 2001). Despite notable shifts in family policies that enhanced maternal labor force participation considerably, such as introducing a paid leave quota for fathers in the parental leave system and the expansion of childcare facilities, the majority of mothers in Germany work part‐time and still assume the main responsibility for childcare and household chores (Lechevalier, 2019). A study on time use among couples found that mothers still spend substantially more time on household chores (e.g., caring, cooking, and cleaning) than fathers and especially on weekdays compared to weekends (Klünder & Meier‐Gräwe, 2018).

Shortages in the provision of widely available childcare—particularly among very young children—further contribute to the highly gendered division of labor (e.g., Boll & Lagemann, 2019). In 2019, only about 34% of children under three years of age attended childcare compared to about 93% of children aged between 3 and 6 years (Federal Ministry for Family Affairs, Senior Citizens, 2019). Because full‐day school options are not yet implemented throughout Germany, only about 50% of elementary school children attended full‐day school or daycare after school in 2019 (Federal Ministry for Family Affairs, Senior Citizens, 2019). Altogether, the current policy framework inconsistently promotes incentives for both joint and separate spheres, which is echoed by societal work‐care ideologies that approve women's employment, yet also their role as primary caregivers (Grunow et al., 2018).

In addition, country‐specific regulations and rates of the access to and use of WFH arrangements may not only influence gender differences but, more generally, the impact of WFH on parenting. To date, there is no legal right to work from home in Germany and access depends on formal or informal agreements with the employer or supervisor. Official statistics on the prevalence and types of WFH arrangements are also still missing. Based on survey data, Arntz et al. (2020) estimated that only a small share of about 12% of workers in Germany worked from home regularly before the pandemic compared to, for example, about 30% in the Netherlands, Luxembourg, and some Nordic countries. In Germany, this share increased considerably to about 60% of workers who worked from home partly or entirely during the first lockdown in the spring of 2020. Research for the U.S. reported a share of about 50% of workers for that same period (Brynjolfsson et al., 2020).

## METHOD

### Data

We used data from the first wave of the large‐scale, representative German survey “Growing up in Germany,” which was collected via standardized computer‐assisted personal interviews in 2019 (Braun et al., 2021; Kuger et al., 2020), and a COVID‐19 online module gathered from a subgroup of the sample during summer and fall of 2020 (Kuger et al., 2021). The first wave covered a wide range of topics related to child development and families’ well‐being, such as the parent‐child relationship and the socioeconomic circumstances of families including parents’ work conditions. The COVID‐19 follow‐up re‐surveyed a reduced set of indicators from the first wave and collected timely information on respondents’ experiences during the COVID‐19 pandemic.

For the first wave in 2019, a probability sample of 0–32‐year‐old target persons was drawn using information from local population registers. These target persons and other household members in this target age range, or the primary caretaker for minors, were then contacted and interviewed if they consented to take part in the study. The overall response rate was about 21%.^1^ After correcting for the higher inclusion probabilities of target persons in larger households and adjusting for unit nonresponse by applying a combined weight, the sample represented the target population well with respect to age, gender, immigration status, education, and regional factors (Braun et al., 2021). For the COVID‐19 follow‐up, all households that indicated to be willing to participate in another round of data collection after 2019 were contacted again. The response rate among employed parents was about 17%.^2^

### Analytical sample

In 2019, the full sample comprised 6606 parents (59.6% female) of minors, and 1018 of these parents were re‐surveyed for the COVID‐19 follow‐up in 2020 (60.8% female). We restricted our analyses to working parents in dependent employment who were below the legal retirement age (<66 years) in both waves (i.e., inactive, unemployed, or self‐employed parents and those in vocational training were excluded). This resulted in a sample of 4477 parents in 2019 (51.8% female), of which 697 participated in 2020 (52.7% female). Furthermore, information on both parenting dimensions (see below) was only available for children who were at least 3 years of age because reciprocal processes related to parenting (especially for responsive parenting) require certain skills of children, such as language skills. After excluding parents who had only younger children, the sample comprised 4017 parents in 2019 and 635 in 2020. Of those, about 96% of the sample in 2019 and about 98% in 2020 had full information on all study variables. Thus, our analyses are based on 3839 working parents (53.5% female) nested in 2848 households in 2019 and 620 working parents (54.4% female) nested in 520 households in 2020. With regard to family structure, our sample largely represents parents in heterosexual relationships: In 2019, 347 participants were single parents (87.9% female) and seven participants were in same‐sex relationships (five females). Of those, 42 single parents (80.9% female) and none of the parents in same‐sex relationships participated in 2020.

### Measures

*Responsive and harsh parenting*, as the two dependent variables, were measured with a total of two items each in 2019 and 2020. These items focused on the frequency of verbal parent‐child interactions from parents’ perspective (Greenberger et al., 1994; Walper & Grgic, 2013). Responsive parenting was assessed with the following statements: “I talk to my child(ren) about what he/she has experienced” and “I talk to my child(ren) about things that annoy or concern him/her.” Harsh parenting was assessed with these statements: “I quickly become angry when my child(ren) do not do what I say” and “I punish my child(dren) more than they deserve.” All items were measured on a 6‐point Likert scale ranging from 1 “always or almost always” to 6 “never.” Responses were reversed so that higher values represent more frequently reported responsive or harsh parenting. If parents had multiple children in the target age range, items were rated once per parent and, thus, captured parents’ reported overall tendency to act in responsive or harsh ways towards their children. In 2019, however, parents rated the two items for responsive parenting for each child individually. In order to have comparable measures for responsive parenting over time, we transformed ratings for multiple children per parent in 2019 into an average score across children per parent. Based on principal component factor analysis, which confirmed that responsive and harsh parenting were two distinct dimensions, we formed a mean composite score for each dimension and year (see Table A1 in the online appendix for descriptive statistics). We then constructed difference scores (Δ) for each dimension by subtracting the 2019 score from the score in 2020. Table A1 shows that the original items were correlated moderately to strongly within each dimension, while items of one dimension were only weakly correlated with the items from the other dimension. The difference scores for responsive and harsh parenting were also not significantly correlated, indicating that the two dimensions had changed independently of each other. Table 1 shows descriptive statistics of the dependent variables and bivariate tests of gender differences, which indicated that changes in responsive and harsh parenting differed significantly by gender.

**TABLE 1:** Descriptive sample statistics by gender

	Full sample	Mothers	Fathers	Gender differences
Variables	Mean (Standard deviation)/%			*t*‐test/*chi* ^2^‐test
*Model variables*				
Δ Responsive parenting (range: −2.5–3.5)	−.18 (.92)	−.25 (.89)	−.08 (.96)	**
Δ Harsh parenting (range: −2.5–3.0)	.13 (.68)	.19 (.64)	.05 (.73)	***
Gender (1 = female, 0 = male)	56.46	./.	./.	
(Mostly) WFH (1 = yes, 0 = no)	49.58	46.85	53.13	n.s.
Δ WFC (range: −5.0–5.0)	.00 (1.67)	.04 (1.63)	−.05 (1.71)	n.s.
Higher working hours during lockdown (1 = yes, 0 = no)	26.97	26.99	26.94	n.s.
Lower working hours during lockdown (1 = yes, 0 = no)	36.16	39.48	31.85	**
Use of WFH in 2019 (1 = yes, 0 = no)	51.36	45.29	59.23	**
Level of WFC in 2019 (1 “low” – 6 “high”)	2.76 (1.51)	2.65 (1.47)	2.91 (1.56)	n.s.
Employment status (1 = full‐time, 0 = part‐time)	53.74	23.76	92.61	***
Supervisory position (1 = yes, 0 = no)	50.12	36.17	68.20	***
Essential occupation (1 = yes, 0 = no)	26.00	22.80	30.16	**
Education (1 = tertiary, 0 = primary or intermediate)	52.02	49.08	55.82	n.s.
Immigration (1 = Respondent or parent(s) born outside Germany, 0 = all born in Germany)	18.34	18.56	18.05	n.s.
Age (in years; range: 23–61)	43.13 (6.60)	42.69 (6.33)	43.70 (6.90)	**
No. of children				**
1	31.09	37.35	22.97	
2	58.26	53.33	64.65	
3 or more	10.66	9.33	12.38	
Age groups of children				
At least one child 0–2 years (1 = yes, 0 = no)	12.57	7.11	19.65	***
At least one child 3–5 years (1 = yes, 0 = no)	32.90	28.03	39.21	**
At least one child 6–9 years (1 = yes, 0 = no)	33.02	31.88	34.51	n.s.
At least one child 10–11 years (1 = yes, 0 = no)	19.46	19.04	20.02	n.s.
*N*	620	337	283	
*Selection variables of participation in the follow‐up study*				
Age (in years; range: 22–64)	41.94 (7.03)	41.35 (6.74)	42.69 (7.33)	***
Education				***
Primary (CASMIN 1a–1c)	13.44	10.41	17.34	
Intermediate (CASMIN 2a–2c)	51.02	56.00	44.61	
Tertiary (CASMIN 3a–3b)	35.54	33.59	38.05	
Immigration				***
Respondent born outside Germany	18.42	17.11	20.10	
Respondent born in Germany, parent(s) born abroad	8.59	8.33	8.92	
Respondent and parents born in Germany	72.99	74.56	70.98	
Occupation (ISCO‐08 major groups)				***
Manager	6.04	4.69	7.78	
Professional	24.18	22.40	26.46	
Technician or associate professional	25.61	31.21	18.41	
Clerical support worker	12.13	15.70	7.55	
Service or sales worker	10.50	13.94	6.08	
Elementary occupation	4.45	4.62	4.24	
Other occupation	17.08	7.44	29.48	
Supervisory position (1 = yes, 0 = no)	42.48	31.39	56.75	***
No. of children				***
1	35.38	39.75	29.76	
2	49.45	47.51	51.94	
3 or more	15.17	12.74	18.30	
Financial strain				n.s.
Low	76.38	75.93	76.96	
To some extent	14.57	14.75	14.33	
(Very) high	9.05	9.32	8.71	
*N*	3839	2053	1786	

Abbreviations: WFH, working from home; WFC, work‐to‐family conflict.

Weighted data; two‐tailed *t*‐test with unequal variances for continuous variables and *chi* ^2^‐test for categorical variables (both on unweighted data); n.s. = not significant, *p < .05, **p < .01, ***p < .001.

*WFH*, as the first main independent variable, was based on the question of whether parents had worked from home for more than half of their regular working hours during the first lockdown period in the spring of 2020 (1 = yes, 0 = no). If this was the case, parents were grouped into “(mostly) WFH” and, if not, into “(mostly) onsite work.” Table 1 shows that nearly half of the parents in 2020 reported that they had worked (mostly) from home and that this share did not differ significantly between mothers and fathers. Table A2 in the online appendix further indicates only a few sociodemographic differences in parents’ likelihood to work from home in 2020: Highly educated parents, those with preschool or young schoolchildren, and parents with previous experience with WFH in 2019 were more likely to switch to WFH in 2020, whereas parents in technical and other occupations (including service and sales, machine operating, and craft workers) were less likely to work from home for large parts of their working hours. Because previous research on parenting also indicated sociodemographic differences related to parental education and children's age (e.g., Schmeer et al., 2021; Verweij et al., 2021), we used this information for the selection of control variables, which may confound associations between WFH and parenting (see below).

*WFC*, as the second main independent variable, was measured with the following single‐item indicator both before and during the pandemic: “Because of my work, it is difficult for me to fulfill my family responsibilities.” This item captures a global assessment of work‐related conflicts with one's family life from respondents’ perspectives. It has proven to be a solid summary measure of specific qualities of WFC, such as time‐based, strain‐based, or behavior‐based aspects, which are often at the focus of multidimensional constructs (see Min et al., 2021 for an overview). Answers were given on a scale ranging from 1 “totally agree” to 6 “totally disagree” and were reversed in a way that higher values represent higher levels of conflict. We then constructed a difference score (Δ), which ranged from −5 to 5 (see Table 1).

### Analytical strategy

We used the first difference regression framework (Allison, 2009) to predict changes in responsive and harsh parenting and applied Heckman's sample selection method (Heckman, 1976, 1979) to account for selective participation in the COVID‐19 follow‐up. First difference regression is a fixed effects approach, which is based on difference scores (Δ) calculated from two time points and accounts for time‐constant unobserved heterogeneity (Allison, 2009). This method allowed us to study intraindividual change in parenting from 2019 (t1) to 2020 (t2) and to test whether the effect of time‐constant sociodemographic variables varied over the course of the early pandemic. The Heckman method further allowed us to jointly estimate changes in parenting and participation in the COVID‐19 follow‐up, in order to test whether there were common unobserved factors in the two equations. Major surveys conducting data collection during the pandemic tended to suffer from nonresponse bias, presumably, because of two main reasons. First, some groups could be less available or too strained to participate in these studies (Post et al., 2020). Second, online surveys, which were widely used during the pandemic, may be less appealing to or accessible for certain sociodemographic groups (Cornesse et al., 2021). We therefore examined response probabilities based on information collected in 2019 (see Figure A1 in the online appendix) and used these variables as predictors of participation in the selection equation. All regression tables in the results section show an additional estimate *athrho*, which is a transformed parameter of the correlation between the error terms of the selection and outcome equation. This correlation is significantly different from zero in the models for mothers (see Tables 2 and 4), which indicates that changes in parenting were not independent of mothers’ selection into the COVID‐19 follow up and the approach of sample selection correction was justified. Results for the selection equation are shown in the full models presented in the online appendix. All analyses were conducted in Stata (v15.1) using weights that accounted for design effects and nonresponse in the first wave. Although the data from the COVID‐19 follow‐up do not allow for dyadic analyses, the data still contain about 100 couples. We therefore used standard errors and confidence intervals that adjusted for clustering at the household level.

**TABLE 2:** Heckman sample selection models of Δ responsive parenting by gender

	Mothers			Fathers		
	(1)	(2)	(3)	(4)	(5)	(6)
WFH	.16	.16	.15	.14	.14	.13
	(.12)	(.12)	(.12)	(.12)	(.12)	(.12)
Δ WFC		−.07	−.16**		−.07	−.06
		(.04)	(.05)		(.04)	(.06)
WFH # Δ WFC			.22***			−.02
			(.06)			(.07)
*Control variables*						
Higher working hours during lockdown	−.13	−.09	−.08	.07	.12	.12
	(.11)	(.12)	(.11)	(.14)	(.14)	(.14)
Lower working hours during lockdown	−.02	.00	.02	.26	.26	.26
	(.12)	(.12)	(.11)	(.15)	(.15)	(.15)
Use of WFH in 2019	.03	.05	.04	−.08	−.10	−.09
	(.13)	(.13)	(.13)	(.12)	(.12)	(.13)
Level of WFC in 2019	−.04	−.09*	−.09*	.04	−.00	−.00
	(.04)	(.04)	(.04)	(.04)	(.05)	(.05)
Full‐time work	.01	−.00	−.03	.28	.28	.28
	(.11)	(.11)	(.11)	(.21)	(.21)	(.21)
Supervisory position	−.25*	−.22	−.28*	−.34*	−.34	−.34
	(.12)	(.12)	(.12)	(.17)	(.17)	(.17)
Essential occupation	.25*	.26*	.30**	.33**	.33**	.33**
	(.11)	(.11)	(.11)	(.13)	(.13)	(.12)
Tertiary education	−.44**	−.43**	−.45**	−.20	−.20	−.20
	(.15)	(.16)	(.15)	(.17)	(.17)	(.17)
Respondent and/or parent(s) born outside Germany	.27	.25	.32*	.24	.23	.23
	(.16)	(.16)	(.16)	(.18)	(.17)	(.18)
Age	−.00	.00	.00	.03*	.03*	.03*
	(.01)	(.01)	(.01)	(.01)	(.01)	(.01)
No. of children: 1	.00	.02	.01	−.21	−.22	−.23
	(.14)	(.13)	(.13)	(.22)	(.22)	(.23)
No. of children: 3 or more	.44**	.44**	.45**	.40*	.41*	.41*
	(.15)	(.15)	(.15)	(.19)	(.19)	(.19)
At least one child 0–2 years	.10	.13	.09	−.07	−.06	−.06
	(.19)	(.19)	(.19)	(.18)	(.18)	(.18)
At least one child 3–5 years	−.06	−.03	−.03	.13	.15	.15
	(.14)	(.14)	(.14)	(.16)	(.16)	(.16)
At least one child 6–9 years	.03	.07	.10	−.12	−.12	−.12
	(.11)	(.11)	(.11)	(.13)	(.12)	(.13)
At least one child 10–11 years	−.38**	−.35**	−.37**	−.20	−.19	−.19
	(.12)	(.12)	(.12)	(.15)	(.15)	(.15)
Constant	1.29	1.16	1.21	−1.07	−.85	−.85
	(.72)	(.72)	(.69)	(1.03)	(1.06)	(1.07)
*Selection equation of participation in the follow‐up study*						
* athrho *	−.97***	−.95***	−1.00***	−.44	−.48	−.47
	(.26)	(.28)	(.27)	(.47)	(.47)	(.48)
*N* (t1)	2053	2053	2053	1786	1786	1786
*N* (t2)	337	337	337	283	283	283

*Note*: Unstandardized coefficients; robust standard errors (in parentheses); weighted data; ^*^ *p* < .05, ^**^ *p* < .01, ^***^ *p* < .001. WFH = working from home for more than half of the regular working hours during the first lockdown in spring 2020; WFC = work‐to‐family conflict; Δ = difference score. Reference categories: part‐time work; primary or intermediate education; respondent and parents born in Germany; no. of children: 2. Selection equation variables: age; education; immigration; occupational groups; no. of children; financial strain. Full models are presented in Table A3 in the online appendix.

Analyses were conducted in the following steps. For both parenting dimensions, we first ran separate models for mothers and fathers, which tested the main association between WFH and parenting (Hypothesis 1a–1b), the role of WFC (Hypothesis 2), and the interaction between WFH and WFC (Hypothesis 3a–3b). We then estimated combined models for mothers and fathers, which also tested gender differences in these associations (Hypothesis 4–5). We further examined significant interaction effects by calculating predictive margins and average marginal effects (AME) of WFH and gender (calculated as discrete changes from 0 to 1), which we displayed graphically as well. Lastly, we addressed sociodemographic differences in the changes in responsive and harsh parenting compared to the pre‐pandemic year because prior studies documented stronger pandemic‐related effects for certain subgroups, such as highly educated mothers and parents with young children (e.g., Schmeer et al., 2021; Verweij et al., 2021). We also conducted two sets of sensitivity analyses, which examined the relationship between WFH and Δ WFC and probed the robustness of our findings for the subgroup of partnered mothers.

### Controlling for confounders

The first difference method controls for unobserved time‐constant heterogeneity, yet it does not account for time‐varying effects of time‐constant factors or for time‐varying factors (Allison, 2009). Arguably, the COVID‐19 pandemic can be regarded as an exogenous event that individuals could hardly avoid and which reduced the likelihood of self‐selection into WFH during lockdowns (Möhring et al., 2021). However, as shown above (see also Table A2 in the online appendix) and documented by other studies (Arntz et al., 2020; Brynjolfsson et al., 2020; Fana et al., 2020), the availability and use of WFH differed by sociodemographic factors (e.g., education, occupation, and childcare demands) even during the early stages of the pandemic. In addition, sociodemographic groups with higher work or care demands likely experienced stronger pandemic‐related effects on their parenting. We therefore controlled for the following factors that may confound the hypothesized relationships between changes in WFH, WFC, and parenting (see Table 1 for descriptive statistics): whether parents increased or reduced their working hours during the first lockdown in the spring of 2020; reportedly worked in an essential occupation (i.e., occupations that fulfill fundamental needs of societies, such as grocery clerks, policy officers, medical staff, or workers in public transportation); whether they had experience with WFH in 2019, had worked full‐time or part‐time, held a supervisor position; their level of WFC in 2019; their age; whether they had obtained a tertiary degree (based on the CASMIN Educational Classification; Brauns et al., 2003), they or their parents had immigrated to Germany, and the number and ages of their children in the household (Abendroth & Reimann, 2018; Chung & van der Lippe, 2020; Hipp & Bünning, 2021; Kim, 2020; Möhring et al., 2021; Schmeer et al., 2021; Verweij et al., 2021). We also considered parents’ occupation (ISCO‐08 major groups), family structure (single parent vs. two‐parent families), the presence of other adults in the household, and whether the family lived in Eastern or Western Germany. Results were robust to the inclusion or exclusion of these factors, and changes in parenting did also not differ by these characteristics. Thus, we opted for the more parsimonious model specification excluding these characteristics.

## RESULTS

### Responsive parenting

Table 2 presents the results of separate models for mothers and fathers. The first row shows that, contrary to our expectations, switching to WFH was not directly related to changes in responsive parenting from 2019 to 2020. The coefficients were positive, yet not significant for both mothers and fathers. The gender‐separate analyses did therefore not support Hypothesis 1a or 1b, which proposed WFH to be either positively or negatively associated with responsive parenting. Similarly, Hypothesis 2 had to be rejected as well, because Δ WFC was negative (as hypothesized), yet not significantly related to Δ responsive parenting. However, Model 3 (including the interaction between WFH and Δ WFC) indicates an interdependent relationship between the two variables and Δ responsive parenting for mothers. The negative coefficient of Δ WFC indicates that an increase in WFC was associated with a significant decrease in responsive parenting among mothers who had continued to work (mostly) onsite. The positive interaction effect shows that mothers’ change in responsive parenting was affected less negatively by increases in WFC if they had switched to (mostly) WFH. For example, at one standard deviation (SD) below the mean of Δ WFC (−1.59), the association between WFH and Δ responsive parenting was negative, but not significant for mothers, .15 + [.22 × (−1.59)] = −.20, *p* > .05. At one SD above the mean of Δ WFC (1.67), the association between WFH and Δ responsive parenting was positive and significant, .15 + [.22 × 1.67] = .52, *p* < .01, and corresponded to .58 of one SD (.52/.89) for Δ responsive parenting. This means that if mothers’ WFC had notably increased from 2019 to 2020, the associated change in responsive parenting differed by about 50% of its SD between those who had switched to working (mostly) from home and those who had continued to work (mostly) onsite. The magnitude of this association seems quite substantial. Altogether, the results for mothers supported Hypothesis 3a (instead of Hypothesis 3b), which predicted that the negative relationship between WFC and responsive parenting would be attenuated when parents work from home rather than onsite. Yet for fathers, the results did not support either of these hypotheses because the coefficient for this interaction term was small and not significant.

**FIGURE 1:**
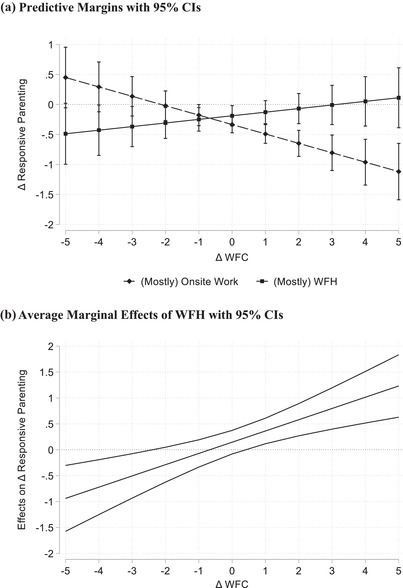
Interaction effect of WFH and Δ WFC on Δ responsive parenting among mothers. (a) Predictive Margins with 95% CIs. (b) Average Marginal Effects of WFH with 95% CIs *Note*: CI = confidence interval; Δ = difference score; positive values on the *y*‐ and *x*‐axis signify increases and negative values decreases in the respective variable; WFH = working from home for more than half of the regular working hours during the first lockdown in the spring of 2020; WFC = work‐to‐family conflict; calculations are based on Model 3 in Table 2.

Figure 1 illustrates the resource effect of WFH predicted in Hypothesis 3a for mothers (based on Model 3 in Table 2). Panel A shows average predicted changes in responsive parenting (predictive margins) stratified by mothers’ use of WFH and conditional on changes in WFC. Positive values on the *y*‐ and *x*‐axis signify increases in responsive parenting and WFC, respectively; negative values indicate decreases, and a value of zero denotes stable levels compared to the previous year. The dashed line indicates that responsive parenting decreased significantly only among mothers who worked (mostly) onsite and, at the same time, experienced similar or even increased levels of WFC. The solid line indicates that if mothers worked (mostly) from home, responsive parenting did not change significantly, irrespective of how their experience of WFC had changed during that same time period.

In addition, Panel B shows a conditional effects plot, which illustrates where in the range of Δ WFC the difference in Δ responsive parenting between WFH and onsite work was significant. The figure displays AME of WFH across the levels of Δ WFC from decreased to increased levels. Confidence intervals that do not contain the value of zero signify significant differences in responsive parenting associated with WFH. The gap in Δ responsive parenting between WFH and onsite work increased significantly for increased levels of Δ WFC (values of 1 or higher). In contrast, for decreased levels of Δ WFC (values of −3 or lower), onsite work was associated with comparatively higher levels of Δ responsive parenting than WFH. However, this latter association has to be interpreted with caution because only a few mothers reported such strong decreases in WFC compared to the pre‐COVID year.

We further found sociodemographic differences in pandemic‐related changes in responsive parenting (see Table 2). For mothers, responsive parenting decreased significantly compared to the pre‐pandemic year among the highly educated and those with supervising responsibilities. This was also true for mothers with children aged 10–11 years. These differences were less pronounced for fathers. However, having three or more children and, surprisingly, working in an essential occupation were associated with an increase in responsive parenting among both mothers and fathers.

Table 3 shows combined models for mothers and fathers, which also tested gender differences in the resource effect of WFH. Results of Models 2 and 3 revealed that the main association between WFH and Δ responsive parenting was positive and significant, even when controlling for changes in WFC. The joint analyses for mothers and fathers thus supported Hypothesis 1a predicting WFH as a resource gain. In order to assess and compare the magnitude of this association, we computed partially standardized coefficients (i.e., dividing the coefficient of WFH by the SD of Δ responsive parenting). The coefficient of .16 for WFH corresponded to .17 SD of Δ responsive parenting, which ranged from −2.5 to 3.5 and had a SD of.92. In other words, the difference in the change of responsive parenting between WFH and onsite work was equivalent to about 17% of the SD of Δ responsive parenting, which corresponds to a small effect size. In absolute terms, responsive parenting decreased only if parents had continued to work onsite (predictive margin of responsive parenting = −.26, *p* < .001), whereas it remained stable if they had switched to WFH (−.09, *p* > .05).

**TABLE 3:** Heckman sample selection models of gender differences in Δ responsive parenting

	(1)	(2)	(3)	(4)	(5)	(6)
Female	−.17	−.16	−.16	−.19	−.20	−.24
	(.11)	(.11)	(.11)	(.11)	(.13)	(.13)
WFH		.16*	.17*	.17*	.13	.12
		(.08)	(.08)	(.08)	(.12)	(.12)
Δ WFC			−.06*	−.11**	−.06*	−.07
			(.03)	(.04)	(.03)	(.05)
WFH # Δ WFC				.10*		−.02
				(.04)		(.06)
WFH # Female					.07	.05
					(.16)	(.16)
Δ WFC # Female						−.06
						(.06)
WFH # Δ WFC # Female						.23**
						(.09)
Constant	.19	.09	.08	.10	.12	.29
	(.60)	(.61)	(.62)	(.62)	(.62)	(.65)
*Selection equation of participation in the follow‐up study*						
* athrho *	−.65**	−.60**	−.58*	−.59*	−.58*	−.64*
	(.22)	(.23)	(.23)	(.23)	(.24)	(.26)
*N* (t1)	3839	3839	3839	3839	3839	3839
*N* (t2)	620	620	620	620	620	620

*Note*: Unstandardized coefficients; standard errors (in parentheses) adjusted for clustering at the level of households [*N* (t1) = 2848; *N* (t2) = 520]; weighted data; ^*^ *p* < .05, ^**^ *p* < .01, ^***^ *p* < .001. WFH = working from home for more than half of the regular working hours during the first lockdown in spring 2020; WFC = work‐to‐family conflict; Δ = difference score. All models control for: changes in working hours; use of WFH in 2019; level of WFC in 2019; full‐time versus part‐time work; supervisory position; working in an essential occupation; education; immigration; age; no. and ages of children. Selection equation variables: age; education; immigration; occupational groups; no. of children; financial strain. Full models are presented in Table A4 in the online appendix.

In addition, results also indicated that WFC was significantly related to reduced levels of responsive parenting in the joint analysis of both genders, which was in line with Hypothesis 2. The fully standardized coefficient was −.11 (−.06 × 1.67/.92), indicating that an increase in Δ WFC of one SD was associated with a decrease of about 11% of the SD of Δ responsive parenting. The magnitude of this relationship appears rather small as well.

Furthermore, results for the combined sample also supported Hypothesis 3a, which predicted that the negative relationship between WFC and responsive parenting would be attenuated when parents work from home rather than onsite. Similar to the pattern of results based on the subsample of mothers, the coefficient for the interaction term in Model 4 was positive and significant, indicating that the difference in Δ responsive parenting between WFH and onsite work increased with increasing levels of Δ WFC. For example, at one SD below the mean of Δ WFC (−1.67), the association between WFH and Δ responsive parenting was basically zero, .17 + [.10 × (−1.67)] = .00, *p* > .05. At one SD above the mean of Δ WFC (1.67), the association between WFH and Δ responsive parenting was positive and significant, .17 + [.10 × 1.67] = .34, *p* < .01, which corresponds to .37 of the SD of Δ responsive parenting (.34/.92). Thus, the magnitude of this coefficient in the joint sample was considerably lower than in the subsample of mothers. Models 5 and 6 provide evidence that, while gender did not generally moderate the relationship between WFH and responsive parenting (Hypothesis 4), the interaction effect of WFH and WFC differed by gender (Hypothesis 5). The coefficient for this three‐way interaction was positive and significant at the 1%‐alpha level and emphasized the obvious differences in the role of WFH and WFC between mothers and fathers found in the stratified analyses. In sum, the results rejected Hypothesis 4, which predicted that the relationship between WFH and parenting would be stronger among mothers compared to fathers. However, results supported Hypothesis 5, which proposed that the impact of the interplay between WFH and WFC on parenting would be more pronounced among mothers compared to fathers.

**FIGURE 2:**
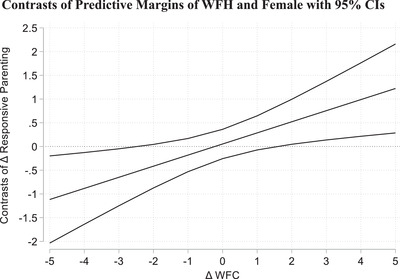
Gender differences in the interaction effect of WFH and Δ WFC on Δ responsive parenting *Note*: CI = confidence interval; Δ = difference score; WFH = working from home for more than half of the regular working hours during the first lockdown in the spring of 2020; WFC = work‐to‐family conflict; calculations are based on Model 6 in Table 3.

For a more detailed examination of gender differences in the joint impact of WFH and WFC on responsive parenting, Figure 2 illustrates this three‐way interaction graphically (based on Model 6 in Table 3). The graph shows differences in the contrasts for WFH (vs. onsite) and being female (vs. male) at different values of Δ WFC. Gender differences in the impact of WFH were significant for substantially increased levels of Δ WFC (1 SD or more), as well as for strongly decreased levels of Δ WFC (about 2 SD). At one SD above the mean of Δ WFC (1.67), the gender difference in the AME of WFH was about .44 and within the 95% CI. This means that the difference in Δ responsive parenting between working (mostly) from home and working (mostly) onsite was larger among mothers compared to fathers. With a magnitude that corresponds to almost half of the SD of Δ responsive parenting (.44/.92), this difference was quite sizeable. In contrast, if WFC had strongly decreased compared to the pre‐pandemic year, the difference in Δ responsive parenting between WFH and onsite work was smaller among mothers compared to fathers. However, as noted before, decreases in WFC of 2 SD or more were observed only for a few parents, which is why we refrain from interpreting this finding.

### Harsh parenting

Results for harsh parenting differed from those for responsive parenting. Table 4 presents separate models for mothers and fathers. Models 1–3 show negative and significant associations between WFH and Δ harsh parenting among mothers. This was in line with Hypothesis 1a, which anticipated WFH to be a resource gain. The coefficient of −.16 for WFH corresponded to .25 SD of Δ harsh parenting, which ranged from −2.5 to 2.5 and had a SD of .64 for mothers. This means that mothers’ change in harsh parenting differed by about 25% of a SD between those who had switched to working (mostly) from home compared to those who had continued to work (mostly) onsite. The magnitude of this relationship was moderate. In absolute terms, harsh parenting increased only among mothers who had continued to work (mostly) onsite (predictive margin of harsh parenting = .26, *p* < .001), whereas it remained stable among those who had switched to working (mostly) from home (.10, *p* > .05). In contrast, the association for fathers was small and not significant and did not support either of the competing Hypotheses 1a or 1b. In addition, Hypothesis 2 had to be rejected for both mothers and fathers, because Δ WFC was positively, yet not significantly related to Δ harsh parenting. Models 3 and 6 further show that the interactions between WFH and Δ WFC were also not significant. Thus, these results prompted us to reject both of the two competing Hypotheses 3a and 3b as well.

**TABLE 4:** Heckman sample selection models of Δ harsh parenting by gender

	Mothers			Fathers		
	(1)	(2)	(3)	(4)	(5)	(6)
WFH	−.17*	−.16*	−.16*	−.00	−.00	−.00
	(.08)	(.08)	(.08)	(.13)	(.13)	(.13)
Δ WFC		.03	.06		.07	.07
		(.03)	(.04)		(.04)	(.05)
WFH # Δ WFC			−.06			.00
			(.05)			(.05)
*Control variables*						
Higher working hours during lockdown	−.01	−.03	−.03	.05	.00	.00
	(.09)	(.09)	(.09)	(.11)	(.11)	(.11)
Lower working hours during lockdown	−.01	−.02	−.03	.19	.20	.20
	(.08)	(.08)	(.08)	(.13)	(.13)	(.13)
Use of WFH in 2019	.10	.09	.09	−.15	−.13	−.13
	(.08)	(.08)	(.08)	(.11)	(.11)	(.11)
Level of WFC in 2019	.01	.03	.03	−.05	−.00	−.00
	(.03)	(.03)	(.03)	(.03)	(.03)	(.03)
Full‐time work	.00	.01	.02	.00	.01	.01
	(.10)	(.10)	(.10)	(.13)	(.12)	(.12)
Supervisory position	.08	.07	.09	.15	.14	.14
	(.09)	(.09)	(.09)	(.11)	(.11)	(.11)
Essential occupation	.03	.03	.02	.04	.04	.04
	(.09)	(.08)	(.08)	(.12)	(.12)	(.12)
Tertiary education	−.30**	−.30**	−.30**	.00	.00	.00
	(.10)	(.10)	(.09)	(.11)	(.11)	(.11)
Respondent and/or parent(s) born outside Germany	.33*	.34*	.33*	.20	.21	.21
	(.14)	(.14)	(.14)	(.15)	(.16)	(.16)
Age	−.00	−.01	−.01	.01	.01	.01
	(.01)	(.01)	(.01)	(.01)	(.01)	(.01)
No. of children: 1	.10	.09	.09	.21	.23	.23
	(.10)	(.10)	(.10)	(.16)	(.16)	(.16)
No. of children: 3 or more	−.04	−.04	−.04	−.30*	−.31*	−.31*
	(.10)	(.10)	(.10)	(.13)	(.14)	(.14)
At least one child 0–2 years	.18	.17	.18	.42*	.41*	.41*
	(.14)	(.14)	(.14)	(.18)	(.18)	(.18)
At least one child 3–5 years	.10	.08	.08	.19	.17	.17
	(.12)	(.12)	(.12)	(.13)	(.12)	(.12)
At least one child 6–9 years	.26**	.24**	.23*	.12	.12	.12
	(.09)	(.09)	(.09)	(.11)	(.11)	(.11)
At least one child 10–11 years	−.05	−.06	−.05	.21	.20	.20
	(.09)	(.09)	(.09)	(.15)	(.14)	(.14)
Constant	1.12*	1.21**	1.20**	−.56	−.75	−.75
	(.45)	(.46)	(.46)	(.67)	(.72)	(.72)
*Selection equation of participation in the follow‐up study*						
* athrho *	−.97***	−1.01***	−1.01***	−.10	−.08	−.08
	(.23)	(.23)	(.23)	(.38)	(.41)	(.41)
*N* (t1)	2053	2053	2053	1786	1786	1786
*N* (t2)	337	337	337	283	283	283

*Note*: Unstandardized coefficients; robust standard errors (in parentheses); weighted data; ^*^ *p* < .05, ^**^ *p* < .01, ^***^ *p* < .001. WFH = working from home for more than half of the regular working hours during the first lockdown in the spring of 2020; WFC = work‐to‐family conflict; Δ = difference score. Reference categories: part‐time work; primary or intermediate education; respondent and parents born in Germany; no. of children: 2. Selection equation variables: age; education; immigration; occupational groups; no. of children; financial strain. Full models are presented in Table A5 in the online appendix.

However, some changes in harsh parenting related to sociodemographic differences are worth highlighting. Harsh parenting increased particularly among parents of young children. Specifically, fathers of toddlers (aged 0–2 years in 2019) and mothers of young school children (aged 6–9 years) reported significantly higher levels of harsh parenting. In addition, harsh parenting also increased among migrant mothers and decreased among highly educated mothers (see Table 4).

The combined models of parents’ changes in harsh parenting also revealed fundamentally different results than those for responsive parenting. The first row in Table 5 indicates that mothers reported higher levels of harsh parenting than fathers during compared to before the pandemic. The difference corresponded to .28 SD of Δ harsh parenting, which ranged from −2.5 to 3 and had a SD of .68 in the combined sample. In absolute terms, harsh parenting had increased only among mothers (predictive margin of harsh parenting = .21, *p* < .001), whereas it had remained stable among fathers (.02, *p* > .05). This gender difference remained significant and increased even slightly after controlling for the use of WFH and changes in WFC, which were not associated with changes in harsh parenting. The two‐way and three‐way interactions tested in Models 4–6 were not significant either. In sum, the results of the combined models for harsh parenting rejected all hypotheses.

**TABLE 5:** Heckman sample selection models of gender differences in Δ harsh parenting

	(1)	(2)	(3)	(4)	(5)	(6)
Female	.19*	.19*	.19*	.19*	.22*	.23*
	(.09)	(.09)	(.09)	(.09)	(.10)	(.11)
WFH		−.08	−.08	−.08	−.04	−.04
		(.07)	(.07)	(.07)	(.10)	(.10)
Δ WFC			.04	.06	.04	.07
			(.02)	(.03)	(.02)	(.04)
WFH # Δ WFC				−.03		.01
				(.04)		(.05)
WFH # Female					−.07	−.06
					(.12)	(.12)
Δ WFC # Female						−.02
						(.05)
WFH # Δ WFC # Female						−.07
						(.07)
Constant	−.06	−.03	−.01	−.00	−.03	−.12
	(.53)	(.51)	(.51)	(.50)	(.51)	(.51)
*Selection equation of participation in the follow‐up study*						
* athrho *	−.33	−.36	−.39	−.39	−.40	−.38
	(.27)	(.25)	(.25)	(.25)	(.26)	(.26)
*N* (t1)	3839	3839	3839	3839	3839	3839
*N* (t2)	620	620	620	620	620	620

*Note*: Unstandardized coefficients; standard errors (in parentheses) adjusted for clustering at the level of households [*N* (t1) = 2848; *N* (t2) = 520]; weighted data; ^*^ *p* < .05, ^**^ *p* < .01, ^***^ *p* < .001. WFH = working from home for more than half of the regular working hours during the first lockdown in spring 2020; WFC = work‐to‐family conflict; Δ = difference score. All models control for: changes in working hours; use of WFH in 2019; level of WFC in 2019; full‐time versus part‐time work; supervisory position; working in an essential occupation; education; immigration; age; no. and ages of children. Selection equation variables: age; education; immigration; occupational groups; no. of children; financial strain. Full models are presented in Table A6 in the online appendix.

### Sensitivity analyses

We conducted two sets of sensitivity analyses to probe the robustness of our results. First, because several studies found a direct link between WFH and increased levels of WFC (see Chung & van der Lippe, 2020 for an overview), we tested this relationship for mothers and fathers. Table A7 in the online appendix shows three different models. The first model only included parents’ use of WFH during the first lockdown in 2020. The second model additionally controlled for sociodemographic differences. The third model further accounts for parents’ use of WFH in 2019 (i.e., before the onset of the pandemic). All models indicated that working (mostly) from home in 2020 was not significantly related to changes in WFC from 2019 to 2020. However, Model 3 shows a positive and significant association between WFH in 2019 and Δ WFC among mothers, whereas this relationship was negative and not significant among fathers. Pre‐pandemic research provided evidence that WFH was often used for supplemental work during overtime and tended to intensify WFC even further, particularly among mothers who, on average, still shoulder the large part of care work (Kim et al., 2020; Ojala et al., 2014). Thus, a small group of mothers may have continued to use WFH for supplemental work during the pandemic and therefore experienced increases in WFC.

The second sensitivity test concerned the analytical sample, which included both partnered and single parents. Because single parents, who are in most cases women, tend to shoulder care demands on their own, whereas cohabiting parents can support each other and manage parenting responsibilities together (Nomaguchi & Milkie, 2020), our hypothesized relationships may be stronger among single compared to partnered parents. We therefore excluded single parents and re‐ran all models for a reduced sample that included cohabiting parents only. Table A8 in the online appendix shows all models for partnered mothers (results for fathers would be almost identical because there were only very few single fathers in the sample), and Tables A9 and A10 show combined models of gender differences between cohabiting mothers and fathers. Overall, results indicated very similar associations compared to the full analytical sample. However, the magnitude of the three‐way interaction (which corresponded to .23 of the SD of Δ responsive parenting) was somewhat smaller than in the full sample. In addition, the main effect of WFH was also slightly reduced and no longer significant. Taken together, the impact of WFH and its interaction with WFC may be even stronger among single than cohabiting parents, yet further research is needed to compare these groups more thoroughly.

## DISCUSSION

After the onset of the COVID‐19 pandemic, many workplaces introduced or expanded WFH options allowing working parents to reorganize work and care, and to remain gainfully employed while compensating for reduced childcare and closed schools during lockdowns (Arntz et al., 2020). Drawing on contextual models of parenting (Belsky, 1984; Belsky & Jaffee, 2006; Bronfenbrenner, 1999; Bronfenbrenner & Morris, 2006), demands‐resources approaches (Bakker & Demerouti, 2007; Demerouti et al., 2001; Voydanoff, 2005a, 2008), and role‐conflict theory (Greenhaus & Beutell, 1985), we hypothesized that WFH as the new normal for many employees during the pandemic may have implied a resource gain or drain for parent‐child interactions. We further expected any potential benefits or burdens associated with WFH to vary by changes in parents’ levels of WFC and to differ by gender.

Based on German survey data gathered before and during early stages of the pandemic, our results rejected the hypothesis of WFH as a resource drain and provided some evidence for the alternative hypothesis of WFH as a resource gain. More specifically, WFH was positively related to responsive parenting in the joint analyses of mothers and fathers, whereas it was negatively related to harsh parenting among mothers only. However, WFH was not associated with actual increases in responsive parenting or decreases in harsh parenting compared to the pre‐pandemic year, but rather buffered pandemic‐related declines in the quality of parent‐child interactions that emerged for those who continued to work mostly or exclusively onsite. Our findings contradict research theorizing WFH as a boundary‐spanning demand (Voydanoff, 2005a, 2005b), and support arguments emphasizing that WFH, if implemented as formal arrangements, can also represent a resource for parents (Kim, 2020; Kim et al., 2020). WFH during the pandemic indeed seemed to increase parents’ schedule control (Allen et al., 2014) and enabled them to organize work and care responsibilities more freely (Kossek et al., 2006). In turn, the resulting gains in time and emotional resources associated with WFH seemed to enable parents—particularly mothers—to maintain their level of responsive parenting, while it prevented them from using harsher behaviors.

Moreover, our results provided partial evidence that the resource gain related to WFH was robust against rising levels of WFC. Responsive parenting was less negatively affected by increases in WFC if parents had switched to WFH. For parents who had continued to work onsite, increases in WFC during the pandemic were associated with significant decreases in responsive parenting compared to the pre‐pandemic year. This adverse impact of WFC was canceled out when parents worked (mostly) from home, which is in line with the job‐demands‐resources model (Bakker & Demerouti, 2007; Demerouti et al., 2001). WFH options during the pandemic appeared to have eased parents’ exposure to high levels of WFC by allowing them to respond to their children's needs immediately, even in situations with a high load of work demands. As a result, parents’ use of responsive behavior in interactions with their children appeared to be less susceptible to fluctuations in WFC when they worked from home rather than onsite. In contrast, results for harsh parenting neither revealed a spillover effect of WFC nor provided evidence for an interaction between WFH and WFC. Although the direction of the relationships was in line with our resource‐gain hypothesis indicating a buffering effect of WFH, the associations were small in size and not significant.

Gender comparisons further revealed remarkable differences in these relationships between mothers and fathers in our sample. WFH‐related resource gains for parenting emerged only among mothers, whereas fathers’ parenting remained largely unaffected by changes in their work situation. Interaction analyses further indicated that, although the relationship between WFH and parenting did not generally differ between mothers and fathers, gender differences in the association between WFH and responsive parenting were significantly more pronounced if parents had experienced increased levels of WFC during the pandemic. These findings suggest gendered implications of WFH arrangements for parenting, which are in line with previous work on the gendered division of care work in heterosexual couple relationships. Studies conducted before and during the pandemic reported that mothers who worked from home were particularly likely to maintain or extend their time spent on childcare (Del Boca et al., 2020; Hank & Steinbach, 2021; Hipp & Bünning, 2021; Powell & Craig, 2015; Shockley et al., 2021; Zoch et al., 2021).

Due to the stronger overlap of work and care roles among mothers compared to fathers, irrespective of family structure, WFH options may have been particularly useful for mothers to remain emotionally available for their children and maintain responsive parent‐child relationships (see also Matias et al., 2017). This may have become even more important if the increased role overlap was caused by rising work demands (see also Adisa et al., 2021). Yet despite WFH‐related resource gains for mothers’ interactions with their children, the greater dissolution of spatial and temporal boundaries between work and family during the pandemic (Lantsoght et al., 2021; Otonkorpi‐Lehtoranta et al., 2021; Salin et al., 2020) has also shown to reduce psychological well‐being among mothers especially (Hipp & Bünning, 2021; Huebener et al., 2021). At the same time, fathers did not seem to benefit from WFH compared to onsite work to engage in responsive interactions with their children more frequently. Because our sample largely represents parents in heterosexual relationships, further research on heterogeneities by family structure is needed. Previous work found evidence that WFH during the pandemic represented a parenting resource for single mothers and partnered lesbian mothers as well (Craig & Churchill, 2021; Goldberg, McCormick, & Virginia, 2021; Sánchez‐Mira et al., 2021). Our results may therefore apply to a variety of family forms. Future work should attempt to systematically compare the effects of WFH by family structure and its intersection with other sociodemographic characteristics across different contexts.

Gender differences in the role of WFH and its interplay with WFC may be more pronounced in institutional and cultural contexts that provide institutional support for gendered separate spheres, such as in Germany, where our study was set. For example, Kurowska (2020) found that, for a sample of parents in Poland, WFH was associated with increased time for childcare and household chores only among mothers. Yet in a sample of parents in Sweden, which is a country with more egalitarian gender norms and family policies, time for childcare and domestic duties increased among both mothers and fathers. Thus, in countries where social policies and cultural norms promote the involvement of fathers in childcare more strongly, WFH may have benefits for father‐child relationships as well.

Some similarities across genders are worth noting as well. Contrary to our expectations, changes in WFC were not related to overall changes in harsh parenting among mothers or fathers, and we found no evidence for the expected interaction effect of WFH and WFC on harsh parenting either. This finding is partly at odds with pre‐pandemic research, which showed that higher levels of WFC were associated with harsher parenting (Cooklin et al., 2015; Haines et al., 2020; Hess & Pollmann‐Schult, 2020). One explanation could be that family‐related factors, such as increased relationship conflicts resulting from disruptions of parents’ and children's routines (Schmid et al., 2021), were more important than work‐related factors in explaining changes in harsh parenting (Prime et al., 2020). Another possible explanation is that, as has been shown for other risk factors associated with poorer parenting (Burchinal et al., 2008), levels of WFC must first exceed a certain threshold in order to affect parenting. However, this threshold may not have been reached, which could be related to the timing of data collection for our study (see limitations below).

### Limitations and future research

Our study has several limitations. First, data collection for the COVID‐19 follow‐up took place throughout the summer and fall months of 2020 when the pandemic situation was quite mild in Germany with an average of 7200 new COVID‐19 infections per week (Robert Koch Institute, 2020). In contrast, a surge of cases was documented in the early spring months, as well as toward the end of 2020, in Germany and many other countries. Measures to contain the spread of COVID‐19 varied between Germany's federal states. However, many childcare centers and schools were closed in the early months of 2020. During the summer and fall of 2020, childcare often continued in a restricted regular mode (e.g., shortened opening hours) and schools continued teaching in a combined mode of in‐person and distance learning. However, because the number of infections fell over the summer and fall months, childcare and schools gradually returned to in‐person learning (Reimer et al., 2021). Thus, closures of childcare centers and schools should not have had a strong effect on the patterns of our findings.

It could further be the case that our results on changes in parenting and gender disparities in these changes were more pronounced in different countries with larger outbreaks during these months or if data collection would have taken place in the spring or winter months of 2020. After the first surge of cases in the early spring months of 2020, parents may have already been used to dealing with social‐distancing measures related to the pandemic and developed routines to juggle work and family demands under these new circumstances. This, in turn, could have dampened the impact on changes related to both responsive and harsh parenting, yet also provide a more realistic outlook on the potential post‐pandemic implications of WFH.

Second, our sample was too small to differentiate and contrast distinct sociodemographic characteristics (e.g., race, ethnicity, single‐parent status, education) to study intersections with gender. However, our results revealed that responsive parenting decreased significantly among highly‐educated mothers and those with supervising responsibilities, whereas harsh parenting increased particularly among mothers of young school children. Because parents with very young children have shown to be particularly challenged during the pandemic (Schmeer et al., 2021), the impact of WFH and increased work demands could be more pronounced for this subgroup. Similarly, parents with different levels of educational attainment may also have coped differently with increased demands in both the work and family spheres during the pandemic (Martucci, 2021; Verweij et al., 2021). Future research based on larger samples or targeted data collection is therefore needed to study how intersections of sociodemographic characteristics modify the impact of the work context on parenting. Research adopting an intersectionality approach could also shed light on the underlying mechanisms within and outside couple relationships.

Third, family forms are diverse, yet our study was not able to differentiate between, for example, heterosexual and same‐sex parents because of very low case numbers for same‐sex parents in the probability sample. We were therefore not able to unpack how the interplay between work conditions and gender may also intersect with sexuality to affect parenting. Quantitative research on this intersection may therefore rely on nonprobability samples collected through social media platforms and snowball sampling. Our data did also not allow for the analyses of dyadic data (e.g., with regard to parents’ division of domestic labor) due to low case numbers for couples. It could be the case that the use of more responsive or harsher parenting is dependent on spousal support (Oppermann et al., 2021). For example, harsher parenting may increase if spousal support from the other parent decreases, which could be the case if, for instance, only one parent can work from home. Investigating the dynamics related to WFH and parenting among dual‐earner couples is an important avenue of research on gender equity, which has important implications beyond the immediate effects of the COVID‐19 pandemic.

Lastly, we restricted our sample to parents who were in dependent employment both in 2019 and 2020 because our study focused on the role of WFH and its interplay with changes in WFC. This resulted in the exclusion of parents, particularly mothers, who stopped working completely in response to increased care demands during the pandemic. Although this was beyond the scope of our work, research on this group is needed as well in order to target risk groups for social policies that support these mothers to remain gainfully employed.

### Practical and policy implications

The expansion of WFH after the onset of the COVID‐19 pandemic is likely to be a permanent change in workers’ routines (Kramer & Kramer, 2021). Different studies estimated that about four out of ten jobs could be partly or fully carried out at home (e.g., Arntz et al., 2020 for Germany; Dingel & Neiman, 2020 for the U.S.). In addition, Germany's new government formed in December 2021 agreed to facilitate WFH for employees by law. Formal agreements defining the scope and use of WFH may indeed promote resource gains for parents to engage with their children in responsive and enriching interactions (Kim, 2020). In pre‐pandemic times, WFH was often used to complete supplemental work during overtime hours rather than during regular working hours, which resulted in higher levels of WFC for parents (Chung & van der Lippe, 2020). The fact that our study did not confirm this association may suggest that formal WFH arrangements allow parents more discretion and long‐term planning in the process of managing their work and caregiving roles (Kelly et al., 2008).

However, because WFH tends to reinforce gender gaps in childcare and parenting, formal arrangements alone may not be sufficient to manage work‐family boundaries effectively. First, affordable institutional childcare for preschool children and supervision for young school children after school hours are essential for mothers to reduce additional childcare hours and to focus on their job when working from home (Martucci, 2021). The lack of external childcare was already a major obstacle to women's (full‐time) employment before the pandemic (Boll & Lagemann, 2019), and it was also mostly women who were cutting back on working hours during the pandemic to shoulder the additional burden of care work and homeschooling (Collins et al., 2021; Petts et al., 2021). Second, workplace cultures that are still oriented towards the male breadwinner as the *ideal worker* (Acker, 1990; Williams, 2000) need to change. Fathers are still hesitant to use flexible work arrangements for family reasons (Sullivan & Lewis, 2001; Sullivan & Smithson, 2007) and if they do, they tend to work longer hours when they use them (Lott & Chung, 2016). Engaging management staff, employees, and employee representatives in work redesign processes that create a commitment to new work routines, which are shared at all organizational levels, has been a promising approach to changing the ideal worker norm (Kossek & Ollier‐Malaterre, 2020). Third and at the family level, couples may also need to develop strategies for supporting each other and balancing both partners’ work demands and care responsibilities (Shockley et al., 2021). Family support counselors, as well as human resource managers, could provide guidance on different models and strategies for dual‐earner couples and families, such as working from home on alternating days.

Even though the demand for and use of WFH has increased substantially in response to social distancing measures related to the COVID‐19 pandemic, access to WFH will likely remain limited to occupational groups that rely mostly on the use of computers or other mobile devices, which tend to be groups of higher socioeconomic status (Arntz et al., 2019; Brynjolfsson et al., 2020; Dingel & Neiman, 2020; Fana et al., 2020). Thus, social disparities related to occupational groups and individuals’ socioeconomic status, which often intersect with other factors (e.g., family structure or ethnicity), became more visible during the pandemic and may even increase in the future (Settersten et al., 2020). Those groups may be particularly at risk of having high work demands spill over on their parenting routines, which can, in turn, diminish the well‐being of children and families as a whole (Chung et al., 2020; Nomaguchi & Milkie, 2020; Prime et al., 2020; Schmeer et al., 2021). Labor market and family policies that strengthen the position of these groups, lower their work stress and levels of WFC, and increase their ability to access better‐paying and more qualified jobs with options to work from home, could help to improve the situation short‐ and long‐term.

## CONCLUSION

Drawing on German survey data gathered before and during the pandemic, our study contributes to much‐needed research on the potential benefits and risks of WFH for parent‐child interactions. The strength of our study is its timely focus on the expansion of flexible work arrangements during the COVID‐19 pandemic, yet the salience of this new normal for parts of the workforce and its ripple effects for families are likely to transcend the pandemic. Taken together, our results suggest that parent‐child interactions may have suffered less if parents—particularly mothers—were able to switch to WFH during the pandemic. This supports the notion that flexible work arrangements can allow parents to organize work and care duties more freely and to their benefit (Kim, 2020; Kim et al., 2020). Findings further suggest that WFH was particularly useful for mothers to engage in more responsive parenting and less harsh parenting, whereas fathers’ parenting remained largely unaffected by changes in their work situation. These gendered patterns highlight the persistent and potentially deepening division of care work within families during the pandemic and are likely upheld by traditional gender norms ingrained in welfare state institutions (Grunow et al., 2018) and workplace cultures (Acker, 1990). Yet strengthening fathers in their role as parents could not only improve father‐child relationships and benefit children's development (Schoppe‐Sullivan & Fagan, 2020), but it may also help mothers to focus on their careers (Martucci, 2021). Furthermore, policymakers and family support services will need to increase their efforts to ease the strains of those working in jobs that cannot be performed from home. This could assist these parents in maintaining responsive and healthy relationships with their children, even under unprecedented circumstances like the COVID‐19 pandemic, which increased the demands on parents in so many ways.

## CONFLICT OF INTEREST

The authors have no conflicts of interest or competing interests to disclose that are relevant to the content of this article.

## Supporting information

Supporting Information

## Footnotes

1 The response rate was calculated based on the most conservative of different possible definitions as described by The American Association for Public Opinion Research (2016). More specifically, the rate was calculated as the number of complete interviews at the household level (*N* = 6355) divided by the gross sample, which refers to the number of household addresses drawn from the population registers (*N* = 30186). Moreover, the gross sample was chosen to be about five times larger than the target household sample size (*N* = 6000). This was done to ensure that the target sample size will be reached even though a significant proportion of households may be unavailable, not eligible, or may refuse to participate in the survey. Although not all of those addresses were actually used, the size of the gross sample still affects the response rate: the larger the gross sample (in relation to the target sample), the lower the response rate. In sum, both the definition of the response rate and the calculation of its defining parts need to be taken into account when interpreting and comparing response rates (ibid.).

2 Over the past years, and particularly during the COVID‐19 pandemic, response rates to surveys based on probability samples have declined (Cornesse et al., 2021; National Research Council, 2013). Nonresponse reduces the power in statistical analyses and creates the potential for bias (see Andridge & Valliant, 2022 for an overview). With regard to power, opt‐in web surveys, which have become widely used during the pandemic, may provide an attractive alternative to probability samples because they may be able to realize larger sample sizes by recruiting volunteers through social media platforms and snowball sampling. In contrast, probability samples set up before the pandemic have suffered from panel attrition and power losses. For example, the low response rate to the follow‐up survey used in this study also resulted in comparatively low case numbers, which limit its potential for studying group‐specific changes in parenting (see the discussion section). Yet with regard to bias, panel surveys based on probability sampling have a major advantage over surveys based on nonprobability sampling such as volunteer samples. Because of self‐selection into nonprobability samples, more approachable or motivated individuals are likely to participate in these surveys. Consequently, the composition of the sample and outcomes of interest can differ vastly from those of a probability sample. Probability sampling, in contrast, involves randomization in the selection process, and each unit in the target population has a known probability of being selected into the sample. These probabilities can be used to make inferences about population parameters, and there are techniques to assess and account for potential biases (see analytical strategy).
