# Supplementary material for: Work from home and parenting: Examining the role of work‐family conflict and gender during the COVID‐19 pandemic
Source: J Soc Issues. 2022 Jun 26:10.1111/josi.12509. Online ahead of print. doi: 10.1111/josi.12509 (PMC9349714; doi:10.1111/josi.12509)
Supplement: Supplementary file 1 — Supporting Information [file JOSI-9999-0-s001.docx]

**Work from home and parenting:**

**Examining the role of work-family conflict and gender during the COVID-19 pandemic**

**Online appendix**(Supplementary material)

Content:

Table A1: Pairwise correlations of the main study variables

Table A2: Logistic regression of working (mostly) from home vs. onsite during the first lockdown period in the spring of 2020

Figure A1: Logistic regression models of participation in the COVID-19 follow-up

Table A3: Heckman sample selection models of Δ responsive parenting by gender

Table A4: Heckman sample selection models of gender differences in Δ responsive parenting

Table A5: Heckman sample selection models of Δ harsh parenting by gender

Table A6: Heckman sample selection models of gender differences in Δ harsh parenting

Table A7: Heckman sample selection models of Δ WFC on WFH during and before the COVID-19 pandemic

Table A8: Heckman sample selection models of Δ responsive parenting and Δ harsh parenting among partnered mothers

Table A9: Heckman sample selection models of gender differences in Δ responsive parenting among partnered parents

Table A10: Heckman sample selection models of gender differences in Δ harsh parenting among partnered parents

Table A1: Pairwise correlations of the main study variables

| Indicators | (1) | (2) | (3) | (4) | (5) | (6) | (7) | (8) | (9) | (10) | (11) | (12) | (13) | M (SD) |
| --- | --- | --- | --- | --- | --- | --- | --- | --- | --- | --- | --- | --- | --- | --- |
| (1) Responsive parenting 1 2019 | 1.00 |  |  |  |  |  |  |  |  |  |  |  |  | 5.27 (.93) |
| (2) Responsive parenting 2 2019 | .75*** | 1.00 |  |  |  |  |  |  |  |  |  |  |  | 5.08 (1.07) |
| (3) Responsive parenting 1 2020 | .41*** | .39*** | 1.00 |  |  |  |  |  |  |  |  |  |  | 5.21 (.84) |
| (4) Responsive parenting 2 2020 | .39*** | .41*** | .62*** | 1.00 |  |  |  |  |  |  |  |  |  | 4.80 (1.07) |
| (5) Δ Responsive parenting | -.53*** | -.54*** | .38*** | .42*** | 1.00 |  |  |  |  |  |  |  |  | -.18 (.92) |
| (6) Harsh parenting 1 2019 | -.12** | -.11** | -.20*** | -.16*** | -.06 | 1.00 |  |  |  |  |  |  |  | 2.46 (.90) |
| (7) Harsh parenting 2 2019 | -.12** | -.13** | -.20*** | -.13** | -.03 | .48*** | 1.00 |  |  |  |  |  |  | 1.72 (.78) |
| (8) Harsh parenting 1 2020 | -.01 | -.04 | -.07 | -.03 | -.03 | .48*** | .31*** | 1.00 |  |  |  |  |  | 2.76 (.94) |
| (9) Harsh parenting 2 2020 | .02 | -.01 | -.09* | -.06 | -.08 | .35*** | .48*** | .43*** | 1.00 |  |  |  |  | 1.67 (.78) |
| (10) Δ Harsh parenting | .15*** | .12** | .15*** | .13** | -.00 | -.40*** | -.40*** | .44*** | .36*** | 1.00 |  |  |  | .13 (.68) |
| (11) WFH | .03 | .01 | .07 | .07 | .05 | .06 | -.02 | .00 | -.07 | -.06 | 1.00 |  |  | .50 |
| (12) Δ WFC | -.02 | .02 | -.08 | -.08 | -.08* | .07 | .04 | .15*** | .10* | .09* | .05 | 1.00 |  | .00 (1.67) |
| (13) Female | .25*** | .25*** | .16*** | .19*** | -.09* | -.08* | -.23*** | -.02 | -.13* | .10* | .03 | -.06 | 1.00 | .56 |

*Notes*: Pearson’s correlation coefficients; weighted data; * *p* < .05, ** *p* < .01, *** *p* < .001.

WFH = Working from home for more than half of the regular working hours during the first lockdown in spring 2020; WFC = Work-to-family conflict; Δ = Difference score; M = Mean; SD = Standard deviation.

Responsive parenting 1: “I talk to my child / children about what he / she has experienced.”

Responsive parenting 2: “I talk to my child / children about things that annoy or concern him / her.”

Harsh parenting 1: “I quickly become angry when my child / children do not do what I say.”

Harsh parenting 2: “I punish my child / children more than they deserve.”

Ranges: Responsive parenting 1 2019 (2–6), Responsive parenting 2 2019 (2–6), Responsive parenting 1 2020 (2–6), Responsive parenting 2 2020 (2–6), Δ Responsive parenting (-2.5–3.5), Harsh parenting 1 2019 (1–6), Harsh parenting 2 2019 (1–5), Harsh parenting 1 2020 (1–6), Harsh parenting 2 2020 (1–5), Δ Harsh parenting (-2.5–3.0), WFH (0–1), Δ WFC (-5.0–5.0), Female (0–1).

Table A2: Logistic regression of working (mostly) from home during the first lockdown period in the spring of 2020

|  | (1) | | (2) | |
| --- | --- | --- | --- | --- |
| Female | -0.05 | (0.06) | -0.02 | (0.05) |
| Age | 0.01 | (0.00) | 0.00 | (0.00) |
| Tertiary education | 0.29^***^ | (0.06) | 0.22^***^ | (0.06) |
| Respondent or parent(s) born outside Germany | 0.06 | (0.05) | 0.06 | (0.06) |
| Manager | -0.10 | (0.10) | -0.13 | (0.10) |
| Professional | -0.10 | (0.08) | -0.12 | (0.08) |
| Technician or associate professional | -0.29^***^ | (0.07) | -0.23^**^ | (0.08) |
| Other occupation | -0.42^***^ | (0.08) | -0.30^***^ | (0.08) |
| Essential occupation | -0.03 | (0.04) | -0.02 | (0.04) |
| Supervisory position | -0.03 | (0.04) | -0.04 | (0.04) |
| Full-time work | -0.01 | (0.06) | 0.00 | (0.06) |
| No. of children: 1 | 0.06 | (0.05) | 0.05 | (0.05) |
| No. of children: 3 or more | -0.06 | (0.05) | -0.05 | (0.05) |
| No. of children: 4 or more | -0.01 | (0.08) | -0.03 | (0.07) |
| At least one child 0–2 years | -0.06 | (0.07) | -0.09 | (0.06) |
| At least one child 3–5 years | 0.09 | (0.05) | 0.10^*^ | (0.05) |
| At least one child 6–9 years | 0.10^*^ | (0.04) | 0.08 | (0.04) |
| At least one child 10–11 years | 0.08 | (0.05) | 0.06 | (0.05) |
| Single parent | -0.05 | (0.07) | -0.05 | (0.06) |
| Other grown-ups in household | 0.10 | (0.08) | 0.10 | (0.06) |
| Residence in Eastern Germany | -0.01 | (0.05) | -0.00 | (0.05) |
| Use of WFH in 2019 |  |  | 0.32^***^ | (0.05) |
| Level of WFC in 2019 |  |  | -0.01 | (0.01) |
| *N* | 620 |  | 620 |  |

*Notes*: Average marginal effects (AME); standard errors (in parentheses) adjusted for clustering at the level of households (*N* = 520); weighted data;

^*^ *p* < 0.05, ^**^ *p* < 0.01, ^***^ *p* < 0.001.

WFH = work from home; WFC = work-to-family conflict.

Reference categories: male; primary or intermediate education; respondent and parents born in Germany; clerical support worker; occupation not essential; no supervisory position; part-time work; no. of children: 2; cohabiting; not living with additional grown-ups; residence in Western Germany; no use of WFH in 2019.

Figure A1: Logistic regression of participation in the COVID-19 follow-up

*Notes*: Standard errors adjusted for clustering at the level of households; weighted data; ^*^ *p* < 0.05, ^**^ *p* < 0.01, ^***^ *p* < 0.001. WFH = work from home.

Reference categories: male; intermediate education; respondent and parents born in Germany; clerical support worker; no supervisory position; part-time work; no use of WFH in 2019; no. of children: 2; cohabiting; not living with additional grown-ups; low financial strain; residence in Western Germany.

Table A3: Heckman sample selection models of Δ responsive parenting by gender

|  |  | Mothers |  |  | Fathers |  |
| --- | --- | --- | --- | --- | --- | --- |
|  | (1) | (2) | (3) | (4) | (5) | (6) |
| WFH | 0.16 | 0.16 | 0.15 | 0.14 | 0.14 | 0.13 |
|  | (0.12) | (0.12) | (0.12) | (0.12) | (0.12) | (0.12) |
| Δ WFC |  | -0.07 | -0.16^**^ |  | -0.07 | -0.06 |
|  |  | (0.04) | (0.05) |  | (0.04) | (0.06) |
| WFH # Δ WFC |  |  | 0.22^***^ |  |  | -0.02 |
|  |  |  | (0.06) |  |  | (0.07) |
| *Control variables* |  |  |  |  |  |  |
| Higher working hours during lockdown | -0.13 | -0.09 | -0.08 | 0.07 | 0.12 | 0.12 |
|  | (0.11) | (0.12) | (0.11) | (0.14) | (0.14) | (0.14) |
| Lower working hours during lockdown | -0.02 | 0.00 | 0.02 | 0.26 | 0.26 | 0.26 |
|  | (0.12) | (0.12) | (0.11) | (0.15) | (0.15) | (0.15) |
| Use of WFH in 2019 | 0.03 | 0.05 | 0.04 | -0.08 | -0.10 | -0.09 |
|  | (0.13) | (0.13) | (0.13) | (0.12) | (0.12) | (0.13) |
| Level of WFC in 2019 | -0.04 | -0.09* | -0.09* | 0.04 | -0.00 | -0.00 |
|  | (0.04) | (0.04) | (0.04) | (0.04) | (0.05) | (0.05) |
| Full-time work | 0.01 | -0.00 | -0.03 | 0.28 | 0.28 | 0.28 |
|  | (0.11) | (0.11) | (0.11) | (0.21) | (0.21) | (0.21) |
| Supervisory position | -0.25* | -0.22 | -0.28* | -0.34* | -0.34 | -0.34 |
|  | (0.12) | (0.12) | (0.12) | (0.17) | (0.17) | (0.17) |
| Essential occupation | 0.25* | 0.26* | 0.30** | 0.33** | 0.33** | 0.33** |
|  | (0.11) | (0.11) | (0.11) | (0.13) | (0.13) | (0.12) |
| Tertiary education | -0.44** | -0.43** | -0.45** | -0.20 | -0.20 | -0.20 |
|  | (0.15) | (0.16) | (0.15) | (0.17) | (0.17) | (0.17) |
| Resp. and/or parent(s) born outside Germany | 0.27 | 0.25 | 0.32* | 0.24 | 0.23 | 0.23 |
|  | (0.16) | (0.16) | (0.16) | (0.18) | (0.17) | (0.18) |
| Age | -0.00 | 0.00 | 0.00 | 0.03* | 0.03* | 0.03* |
|  | (0.01) | (0.01) | (0.01) | (0.01) | (0.01) | (0.01) |
| No. of children: 1 | 0.00 | 0.02 | 0.01 | -0.21 | -0.22 | -0.23 |
|  | (0.14) | (0.13) | (0.13) | (0.22) | (0.22) | (0.23) |
| No. of children: 3 or more | 0.44** | 0.44** | 0.45** | 0.40* | 0.41* | 0.41* |
|  | (0.15) | (0.15) | (0.15) | (0.19) | (0.19) | (0.19) |
| At least one child 0–2 years | 0.10 | 0.13 | 0.09 | -0.07 | -0.06 | -0.06 |
|  | (0.19) | (0.19) | (0.19) | (0.18) | (0.18) | (0.18) |
| At least one child 3–5 years | -0.06 | -0.03 | -0.03 | 0.13 | 0.15 | 0.15 |
|  | (0.14) | (0.14) | (0.14) | (0.16) | (0.16) | (0.16) |
| At least one child 6–9 years | 0.03 | 0.07 | 0.10 | -0.12 | -0.12 | -0.12 |
|  | (0.11) | (0.11) | (0.11) | (0.13) | (0.12) | (0.13) |
| At least one child 10–11 years | -0.38** | -0.35** | -0.37** | -0.20 | -0.19 | -0.19 |
|  | (0.12) | (0.12) | (0.12) | (0.15) | (0.15) | (0.15) |
| Constant | 1.29 | 1.16 | 1.21 | -1.07 | -0.85 | -0.85 |
|  | (0.72) | (0.72) | (0.69) | (1.03) | (1.06) | (1.07) |
| *Selection equation of participation in the follow-up study* |  |  |  |  |  |  |
| Age | 0.02** | 0.02** | 0.02** | 0.01 | 0.01 | 0.01 |
|  | (0.01) | (0.01) | (0.01) | (0.01) | (0.01) | (0.01) |
| Primary education | -0.42 | -0.42 | -0.41 | -0.28 | -0.28 | -0.28 |
|  | (0.24) | (0.24) | (0.23) | (0.14) | (0.15) | (0.15) |
| Tertiary education | 0.25** | 0.25** | 0.25** | 0.31** | 0.31** | 0.31** |
|  | (0.09) | (0.09) | (0.09) | (0.12) | (0.12) | (0.12) |
| Respondent born outside Germany | -0.28* | -0.28* | -0.29* | -0.49*** | -0.49*** | -0.49*** |
|  | (0.13) | (0.13) | (0.13) | (0.13) | (0.13) | (0.13) |
| Parent(s) born outside Germany | -0.05 | -0.05 | -0.04 | -0.03 | -0.03 | -0.03 |
|  | (0.15) | (0.15) | (0.15) | (0.18) | (0.17) | (0.17) |
| Manager | -0.05 | -0.04 | -0.06 | -0.37 | -0.37 | -0.37 |
|  | (0.20) | (0.20) | (0.20) | (0.22) | (0.21) | (0.21) |
| Professional | -0.03 | -0.03 | -0.03 | -0.46* | -0.46* | -0.46* |
|  | (0.12) | (0.12) | (0.11) | (0.18) | (0.18) | (0.18) |
| Technician or associate professional | -0.10 | -0.10 | -0.10 | -0.61*** | -0.61*** | -0.61*** |
|  | (0.11) | (0.11) | (0.11) | (0.18) | (0.18) | (0.18) |
| Service or sale worker | -0.11 | -0.11 | -0.11 | -0.52 | -0.50 | -0.50 |
|  | (0.14) | (0.14) | (0.14) | (0.27) | (0.28) | (0.28) |
| Elementary occupation | -0.76** | -0.75** | -0.72** | -0.42 | -0.41 | -0.41 |
|  | (0.25) | (0.25) | (0.25) | (0.31) | (0.31) | (0.31) |
| Other occupation | -0.08 | -0.08 | -0.06 | -0.52** | -0.52** | -0.52** |
|  | (0.16) | (0.16) | (0.16) | (0.19) | (0.19) | (0.19) |
| Supervisory position | 0.07 | 0.07 | 0.07 | 0.21* | 0.21* | 0.21* |
|  | (0.09) | (0.09) | (0.09) | (0.09) | (0.09) | (0.09) |
| No. of children: 1 | -0.09 | -0.09 | -0.09 | -0.34** | -0.34** | -0.34** |
|  | (0.09) | (0.09) | (0.09) | (0.12) | (0.12) | (0.12) |
| No. of children: 3 or more | -0.24* | -0.24* | -0.25* | -0.28** | -0.28** | -0.28** |
|  | (0.10) | (0.10) | (0.10) | (0.10) | (0.10) | (0.10) |
| Financial strain: To some extent | -0.34** | -0.33** | -0.33** | -0.20 | -0.19 | -0.19 |
|  | (0.12) | (0.12) | (0.11) | (0.13) | (0.13) | (0.13) |
| Financial strain: (Very) high | -0.47** | -0.47** | -0.45** | -0.40* | -0.41* | -0.41* |
|  | (0.15) | (0.15) | (0.15) | (0.20) | (0.20) | (0.20) |
| Constant | -1.50*** | -1.50*** | -1.50*** | -0.81* | -0.81* | -0.81* |
|  | (0.29) | (0.29) | (0.29) | (0.32) | (0.32) | (0.32) |
| athrho | -0.97*** | -0.95*** | -1.00*** | -0.44 | -0.48 | -0.47 |
|  | (0.26) | (0.28) | (0.27) | (0.47) | (0.47) | (0.48) |
| *N* (t1) | 2053 | 2053 | 2053 | 1786 | 1786 | 1786 |
| *N* (t2) | 337 | 337 | 337 | 283 | 283 | 283 |

*Notes*: Unstandardized coefficients; robust standard errors (in parentheses); weighted data; ^*^ *p* < 0.05, ^**^ *p* < 0.01, ^***^ *p* < 0.001. WFH = working from home for more than half of the regular working hours during the first lockdown in spring 2020; WFC = work-to-family conflict; Δ = difference score. Reference categories (model): part-time work; primary or intermediate education; respondent and parents born in Germany; no. of children: 2. Reference categories (selection equation): intermediate education; respondent and parents born in Germany; clerical support worker; no. of children: 2; low financial strain.

Table A4: Heckman sample selection models of gender differences in Δ responsive parenting

|  | (1) | (2) | (3) | (4) | (5) | (6) |
| --- | --- | --- | --- | --- | --- | --- |
| Female | -0.17 | -0.16 | -0.16 | -0.19 | -0.20 | -0.24 |
|  | (0.11) | (0.11) | (0.11) | (0.11) | (0.13) | (0.13) |
| WFH |  | 0.16^*^ | 0.17^*^ | 0.17^*^ | 0.13 | 0.12 |
|  |  | (0.08) | (0.08) | (0.08) | (0.12) | (0.12) |
| Δ WFC |  |  | -0.06^*^ | -0.11^**^ | -0.06^*^ | -0.07 |
|  |  |  | (0.03) | (0.04) | (0.03) | (0.05) |
| WFH # Δ WFC |  |  |  | 0.10^*^ |  | -0.02 |
|  |  |  |  | (0.04) |  | (0.06) |
| WFH # Female |  |  |  |  | 0.07 | 0.05 |
|  |  |  |  |  | (0.16) | (0.16) |
| Δ WFC # Female |  |  |  |  |  | -0.06 |
|  |  |  |  |  |  | (0.06) |
| WFH # Δ WFC # Female |  |  |  |  |  | 0.23^**^ |
|  |  |  |  |  |  | (0.09) |
| *Control variables* |  |  |  |  |  |  |
| Higher working hours during lockdown | -0.03 | -0.03 | 0.01 | 0.01 | 0.00 | 0.02 |
|  | (0.09) | (0.09) | (0.09) | (0.09) | (0.09) | (0.09) |
| Lower working hours during lockdown | 0.09 | 0.10 | 0.10 | 0.10 | 0.10 | 0.10 |
|  | (0.09) | (0.09) | (0.09) | (0.09) | (0.09) | (0.09) |
| Use of WFH in 2019 | 0.02 | -0.04 | -0.04 | -0.04 | -0.04 | -0.05 |
|  | (0.08) | (0.09) | (0.09) | (0.09) | (0.09) | (0.09) |
| Level of WFC in 2019 | -0.01 | -0.01 | -0.05 | -0.05 | -0.05 | -0.05 |
|  | (0.03) | (0.03) | (0.03) | (0.03) | (0.03) | (0.03) |
| Full-time work | 0.05 | 0.05 | 0.04 | 0.03 | 0.04 | 0.02 |
|  | (0.10) | (0.10) | (0.10) | (0.10) | (0.10) | (0.10) |
| Supervisory position | -0.29^**^ | -0.27^**^ | -0.25^**^ | -0.27^**^ | -0.26^**^ | -0.30^**^ |
|  | (0.09) | (0.09) | (0.09) | (0.09) | (0.10) | (0.10) |
| Essential occupation | 0.29^***^ | 0.30^***^ | 0.30^***^ | 0.30^***^ | 0.30^***^ | 0.32^***^ |
|  | (0.08) | (0.08) | (0.08) | (0.08) | (0.08) | (0.08) |
| Tertiary education | -0.28^**^ | -0.30^**^ | -0.30^**^ | -0.31^**^ | -0.30^**^ | -0.31^**^ |
|  | (0.10) | (0.10) | (0.11) | (0.11) | (0.11) | (0.11) |
| Resp. and/or parent(s) born outside Germany | 0.30^**^ | 0.28^**^ | 0.26^*^ | 0.28^**^ | 0.26^*^ | 0.30^**^ |
|  | (0.11) | (0.11) | (0.11) | (0.11) | (0.11) | (0.11) |
| Age | 0.02 | 0.02 | 0.02 | 0.02 | 0.02 | 0.01 |
|  | (0.01) | (0.01) | (0.01) | (0.01) | (0.01) | (0.01) |
| No. of children: 1 | -0.03 | -0.04 | -0.05 | -0.04 | -0.05 | -0.04 |
|  | (0.11) | (0.11) | (0.11) | (0.11) | (0.11) | (0.11) |
| No. of children: 3 or more | 0.39^**^ | 0.38^**^ | 0.38^**^ | 0.39^***^ | 0.38^**^ | 0.39^**^ |
|  | (0.12) | (0.12) | (0.12) | (0.12) | (0.12) | (0.12) |
| At least one child 0–2 years | -0.01 | 0.00 | 0.03 | 0.03 | 0.03 | 0.01 |
|  | (0.13) | (0.13) | (0.13) | (0.13) | (0.13) | (0.12) |
| At least one child 3–5 years | 0.04 | 0.02 | 0.05 | 0.05 | 0.05 | 0.05 |
|  | (0.10) | (0.10) | (0.10) | (0.10) | (0.10) | (0.10) |
| At least one child 6–9 years | 0.00 | -0.01 | 0.01 | 0.03 | 0.01 | 0.02 |
|  | (0.09) | (0.09) | (0.08) | (0.09) | (0.08) | (0.08) |
| At least one child 10–11 years | -0.24^*^ | -0.26^**^ | -0.24^*^ | -0.24^*^ | -0.24^*^ | -0.25^*^ |
|  | (0.10) | (0.10) | (0.10) | (0.10) | (0.10) | (0.10) |
| Constant | 0.19 | 0.09 | 0.08 | 0.10 | 0.12 | 0.29 |
|  | (0.60) | (0.61) | (0.62) | (0.62) | (0.62) | (0.65) |
| *Selection equation of participation in the follow-up study* |  |  |  |  |  |  |
| Age | 0.01^**^ | 0.01^**^ | 0.01^**^ | 0.01^**^ | 0.01^**^ | 0.01^**^ |
|  | (0.00) | (0.00) | (0.00) | (0.00) | (0.00) | (0.00) |
| Primary education | -0.31^*^ | -0.31^*^ | -0.31^*^ | -0.31^*^ | -0.31^*^ | -0.30^*^ |
|  | (0.14) | (0.14) | (0.14) | (0.14) | (0.14) | (0.14) |
| Tertiary education | 0.27^***^ | 0.27^***^ | 0.28^***^ | 0.28^***^ | 0.27^***^ | 0.28^***^ |
|  | (0.08) | (0.08) | (0.08) | (0.08) | (0.08) | (0.08) |
| Respondent born outside Germany | -0.36^***^ | -0.36^***^ | -0.36^***^ | -0.36^***^ | -0.36^***^ | -0.36^***^ |
|  | (0.10) | (0.10) | (0.10) | (0.10) | (0.10) | (0.10) |
| Parent(s) born outside Germany | -0.03 | -0.03 | -0.03 | -0.03 | -0.03 | -0.03 |
|  | (0.11) | (0.11) | (0.11) | (0.11) | (0.11) | (0.11) |
| Manager | -0.14 | -0.14 | -0.14 | -0.15 | -0.14 | -0.15 |
|  | (0.14) | (0.14) | (0.14) | (0.14) | (0.14) | (0.14) |
| Professional | -0.17 | -0.17 | -0.17 | -0.17 | -0.17 | -0.17 |
|  | (0.11) | (0.11) | (0.11) | (0.11) | (0.11) | (0.11) |
| Technician or associate professional | -0.25^*^ | -0.24^*^ | -0.24^*^ | -0.24^*^ | -0.24^*^ | -0.24^*^ |
|  | (0.11) | (0.11) | (0.11) | (0.11) | (0.11) | (0.11) |
| Service or sale worker | -0.22 | -0.21 | -0.21 | -0.21 | -0.21 | -0.21 |
|  | (0.13) | (0.13) | (0.13) | (0.13) | (0.13) | (0.13) |
| Elementary occupation | -0.53^**^ | -0.53^**^ | -0.52^*^ | -0.52^*^ | -0.52^*^ | -0.51^*^ |
|  | (0.20) | (0.20) | (0.20) | (0.20) | (0.20) | (0.21) |
| Other occupation | -0.27^*^ | -0.26^*^ | -0.27^*^ | -0.27^*^ | -0.27^*^ | -0.26^*^ |
|  | (0.11) | (0.12) | (0.12) | (0.11) | (0.12) | (0.11) |
| Supervisory position | 0.11 | 0.11 | 0.12 | 0.12 | 0.11 | 0.12 |
|  | (0.06) | (0.06) | (0.06) | (0.06) | (0.06) | (0.06) |
| No. of children: 1 | -0.18^*^ | -0.18^*^ | -0.18^*^ | -0.18^*^ | -0.18^*^ | -0.18^*^ |
|  | (0.08) | (0.08) | (0.08) | (0.08) | (0.08) | (0.08) |
| No. of children: 3 or more | -0.26^***^ | -0.26^***^ | -0.26^***^ | -0.26^***^ | -0.26^***^ | -0.26^***^ |
|  | (0.08) | (0.08) | (0.08) | (0.08) | (0.08) | (0.08) |
| Financial strain: To some extent | -0.28^**^ | -0.29^**^ | -0.28^**^ | -0.28^**^ | -0.28^**^ | -0.28^**^ |
|  | (0.10) | (0.10) | (0.10) | (0.10) | (0.10) | (0.10) |
| Financial strain: (Very) high | -0.46^***^ | -0.47^***^ | -0.47^***^ | -0.47^***^ | -0.47^***^ | -0.46^***^ |
|  | (0.13) | (0.13) | (0.13) | (0.13) | (0.13) | (0.13) |
| Constant | -1.19^***^ | -1.19^***^ | -1.19^***^ | -1.19^***^ | -1.19^***^ | -1.20^***^ |
|  | (0.22) | (0.22) | (0.22) | (0.22) | (0.22) | (0.22) |
| athrho | -0.65^**^ | -0.60^**^ | -0.58^*^ | -0.59^*^ | -0.58^*^ | -0.64^*^ |
|  | (0.22) | (0.23) | (0.23) | (0.23) | (0.24) | (0.26) |
| *N* (t1) | 3839 | 3839 | 3839 | 3839 | 3839 | 3839 |
| *N* (t2) | 620 | 620 | 620 | 620 | 620 | 620 |

*Notes*: Unstandardized coefficients; standard errors (in parentheses) adjusted for clustering at the level of households [*N* (t1) = 2848; *N* (t2) = 520]; weighted data; ^*^ *p* < 0.05, ^**^ *p* < 0.01, ^***^ *p* < 0.001.

WFH = working from home for more than half of the regular working hours during the first lockdown in spring 2020; WFC = work-to-family conflict; Δ = difference score. Reference categories (model): part-time work; primary or intermediate education; respondent and parents born in Germany; no. of children: 2. Reference categories (selection equation): intermediate education; respondent and parents born in Germany; clerical support worker; no. of children: 2; low financial strain.

Table A5: Heckman sample selection models of Δ harsh parenting by gender

|  |  | Mothers |  |  | Fathers |  |
| --- | --- | --- | --- | --- | --- | --- |
|  | (1) | (2) | (3) | (4) | (5) | (6) |
| WFH | -0.17^*^ | -0.16^*^ | -0.16^*^ | -0.00 | -0.00 | -0.00 |
|  | (0.08) | (0.08) | (0.08) | (0.13) | (0.13) | (0.13) |
| Δ WFC |  | 0.03 | 0.06 |  | 0.07 | 0.07 |
|  |  | (0.03) | (0.04) |  | (0.04) | (0.05) |
| WFH # Δ WFC |  |  | -0.06 |  |  | 0.00 |
|  |  |  | (0.05) |  |  | (0.05) |
| *Control variables* |  |  |  |  |  |  |
| Higher working hours during lockdown | -0.01 | -0.03 | -0.03 | 0.05 | 0.00 | 0.00 |
|  | (0.09) | (0.09) | (0.09) | (0.11) | (0.11) | (0.11) |
| Lower working hours during lockdown | -0.01 | -0.02 | -0.03 | 0.19 | 0.20 | 0.20 |
|  | (0.08) | (0.08) | (0.08) | (0.13) | (0.13) | (0.13) |
| Use of WFH in 2019 | 0.10 | 0.09 | 0.09 | -0.15 | -0.13 | -0.13 |
|  | (0.08) | (0.08) | (0.08) | (0.11) | (0.11) | (0.11) |
| Level of WFC in 2019 | 0.01 | 0.03 | 0.03 | -0.05 | -0.00 | -0.00 |
|  | (0.03) | (0.03) | (0.03) | (0.03) | (0.03) | (0.03) |
| Full-time work | 0.00 | 0.01 | 0.02 | 0.00 | 0.01 | 0.01 |
|  | (0.10) | (0.10) | (0.10) | (0.13) | (0.12) | (0.12) |
| Supervisory position | 0.08 | 0.07 | 0.09 | 0.15 | 0.14 | 0.14 |
|  | (0.09) | (0.09) | (0.09) | (0.11) | (0.11) | (0.11) |
| Essential occupation | 0.03 | 0.03 | 0.02 | 0.04 | 0.04 | 0.04 |
|  | (0.09) | (0.08) | (0.08) | (0.12) | (0.12) | (0.12) |
| Tertiary education | -0.30^**^ | -0.30^**^ | -0.30^**^ | 0.00 | 0.00 | 0.00 |
|  | (0.10) | (0.10) | (0.09) | (0.11) | (0.11) | (0.11) |
| Resp. and/or parent(s) born outside Germany | 0.33^*^ | 0.34^*^ | 0.33^*^ | 0.20 | 0.21 | 0.21 |
|  | (0.14) | (0.14) | (0.14) | (0.15) | (0.16) | (0.16) |
| Age | -0.00 | -0.01 | -0.01 | 0.01 | 0.01 | 0.01 |
|  | (0.01) | (0.01) | (0.01) | (0.01) | (0.01) | (0.01) |
| No. of children: 1 | 0.10 | 0.09 | 0.09 | 0.21 | 0.23 | 0.23 |
|  | (0.10) | (0.10) | (0.10) | (0.16) | (0.16) | (0.16) |
| No. of children: 3 or more | -0.04 | -0.04 | -0.04 | -0.30^*^ | -0.31^*^ | -0.31^*^ |
|  | (0.10) | (0.10) | (0.10) | (0.13) | (0.14) | (0.14) |
| At least one child 0–2 years | 0.18 | 0.17 | 0.18 | 0.42^*^ | 0.41^*^ | 0.41^*^ |
|  | (0.14) | (0.14) | (0.14) | (0.18) | (0.18) | (0.18) |
| At least one child 3–5 years | 0.10 | 0.08 | 0.08 | 0.19 | 0.17 | 0.17 |
|  | (0.12) | (0.12) | (0.12) | (0.13) | (0.12) | (0.12) |
| At least one child 6–9 years | 0.26^**^ | 0.24^**^ | 0.23^*^ | 0.12 | 0.12 | 0.12 |
|  | (0.09) | (0.09) | (0.09) | (0.11) | (0.11) | (0.11) |
| At least one child 10–11 years | -0.05 | -0.06 | -0.05 | 0.21 | 0.20 | 0.20 |
|  | (0.09) | (0.09) | (0.09) | (0.15) | (0.14) | (0.14) |
| Constant | 1.12^*^ | 1.21^**^ | 1.20^**^ | -0.56 | -0.75 | -0.75 |
|  | (0.45) | (0.46) | (0.46) | (0.67) | (0.72) | (0.72) |
| *Selection equation of participation in the follow-up study* |  |  |  |  |  |  |
| Age | 0.02^*^ | 0.02^*^ | 0.02^*^ | 0.01 | 0.01 | 0.01 |
|  | (0.01) | (0.01) | (0.01) | (0.01) | (0.01) | (0.01) |
| Primary education | -0.39 | -0.39 | -0.40 | -0.30^*^ | -0.30^*^ | -0.30^*^ |
|  | (0.21) | (0.20) | (0.20) | (0.14) | (0.14) | (0.14) |
| Tertiary education | 0.27^**^ | 0.27^**^ | 0.27^**^ | 0.30^*^ | 0.30^*^ | 0.30^*^ |
|  | (0.09) | (0.09) | (0.09) | (0.12) | (0.12) | (0.12) |
| Respondent born outside Germany | -0.28^*^ | -0.28^*^ | -0.28^*^ | -0.47^***^ | -0.46^***^ | -0.46^***^ |
|  | (0.13) | (0.13) | (0.13) | (0.13) | (0.13) | (0.13) |
| Parent(s) born outside Germany | -0.03 | -0.02 | -0.03 | -0.06 | -0.06 | -0.06 |
|  | (0.17) | (0.17) | (0.17) | (0.18) | (0.18) | (0.18) |
| Manager | -0.18 | -0.19 | -0.19 | -0.39 | -0.39 | -0.39 |
|  | (0.20) | (0.20) | (0.19) | (0.23) | (0.23) | (0.23) |
| Professional | -0.04 | -0.04 | -0.04 | -0.47^*^ | -0.47^*^ | -0.47^*^ |
|  | (0.12) | (0.12) | (0.12) | (0.19) | (0.19) | (0.19) |
| Technician or associate professional | -0.08 | -0.08 | -0.08 | -0.61^**^ | -0.61^**^ | -0.61^**^ |
|  | (0.11) | (0.11) | (0.11) | (0.19) | (0.19) | (0.19) |
| Service or sale worker | -0.11 | -0.11 | -0.11 | -0.59^*^ | -0.59^*^ | -0.59^*^ |
|  | (0.15) | (0.14) | (0.14) | (0.24) | (0.24) | (0.24) |
| Elementary occupation | -0.55 | -0.55 | -0.56^*^ | -0.42 | -0.43 | -0.43 |
|  | (0.28) | (0.28) | (0.28) | (0.37) | (0.37) | (0.36) |
| Other occupation | 0.06 | 0.06 | 0.06 | -0.55^**^ | -0.55^**^ | -0.55^**^ |
|  | (0.16) | (0.16) | (0.16) | (0.18) | (0.18) | (0.18) |
| Supervisory position | 0.09 | 0.09 | 0.09 | 0.21^*^ | 0.21^*^ | 0.21^*^ |
|  | (0.09) | (0.09) | (0.09) | (0.09) | (0.09) | (0.09) |
| No. of children: 1 | -0.08 | -0.08 | -0.08 | -0.34^**^ | -0.34^**^ | -0.34^**^ |
|  | (0.09) | (0.09) | (0.09) | (0.12) | (0.12) | (0.12) |
| No. of children: 3 or more | -0.23^*^ | -0.23^*^ | -0.23^*^ | -0.28^**^ | -0.28^**^ | -0.28^**^ |
|  | (0.10) | (0.10) | (0.10) | (0.10) | (0.10) | (0.10) |
| Financial strain: To some extent | -0.41^***^ | -0.42^***^ | -0.42^***^ | -0.22 | -0.23 | -0.23 |
|  | (0.11) | (0.10) | (0.10) | (0.13) | (0.13) | (0.13) |
| Financial strain: (Very) high | -0.58^***^ | -0.58^***^ | -0.58^***^ | -0.38 | -0.38 | -0.38 |
|  | (0.14) | (0.14) | (0.14) | (0.20) | (0.20) | (0.20) |
| Constant | -1.46^***^ | -1.46^***^ | -1.46^***^ | -0.78^*^ | -0.78^*^ | -0.78^*^ |
|  | (0.28) | (0.28) | (0.28) | (0.32) | (0.32) | (0.32) |
| athrho | -0.97^***^ | -1.01^***^ | -1.01^***^ | -0.10 | -0.08 | -0.08 |
|  | (0.23) | (0.23) | (0.23) | (0.38) | (0.41) | (0.41) |
| *N* (t1) | 2053 | 2053 | 2053 | 1786 | 1786 | 1786 |
| *N* (t2) | 337 | 337 | 337 | 283 | 283 | 283 |

*Notes*: Unstandardized coefficients; robust standard errors (in parentheses); weighted data; ^*^ *p* < 0.05, ^**^ *p* < 0.01, ^***^ *p* < 0.001. WFH = working from home for more than half of the regular working hours during the first lockdown in spring 2020; WFC = work-to-family conflict; Δ = difference score. Reference categories (model): part-time work; primary or intermediate education; respondent and parents born in Germany; no. of children: 2. Reference categories (selection equation): intermediate education; respondent and parents born in Germany; clerical support worker; no. of children: 2; low financial strain.

Table A6: Heckman sample selection models of gender differences in Δ harsh parenting

|  | (1) | (2) | (3) | (4) | (5) | (6) |
| --- | --- | --- | --- | --- | --- | --- |
| Female | 0.19^*^ | 0.19^*^ | 0.19^*^ | 0.19^*^ | 0.22^*^ | 0.23^*^ |
|  | (0.09) | (0.09) | (0.09) | (0.09) | (0.10) | (0.11) |
| WFH |  | -0.08 | -0.08 | -0.08 | -0.04 | -0.04 |
|  |  | (0.07) | (0.07) | (0.07) | (0.10) | (0.10) |
| Δ WFC |  |  | 0.04 | 0.06 | 0.04 | 0.07 |
|  |  |  | (0.02) | (0.03) | (0.02) | (0.04) |
| WFH # Δ WFC |  |  |  | -0.03 |  | 0.01 |
|  |  |  |  | (0.04) |  | (0.05) |
| WFH # Female |  |  |  |  | -0.07 | -0.06 |
|  |  |  |  |  | (0.12) | (0.12) |
| Δ WFC # Female |  |  |  |  |  | -0.02 |
|  |  |  |  |  |  | (0.05) |
| WFH # Δ WFC # Female |  |  |  |  |  | -0.07 |
|  |  |  |  |  |  | (0.07) |
| *Control variables* |  |  |  |  |  |  |
| Higher working hours during lockdown | 0.02 | 0.03 | 0.00 | 0.00 | 0.00 | -0.00 |
|  | (0.07) | (0.07) | (0.07) | (0.07) | (0.07) | (0.07) |
| Lower working hours during lockdown | 0.10 | 0.10 | 0.10 | 0.10 | 0.10 | 0.09 |
|  | (0.07) | (0.07) | (0.07) | (0.07) | (0.07) | (0.07) |
| Use of WFH in 2019 | -0.06 | -0.03 | -0.03 | -0.03 | -0.03 | -0.02 |
|  | (0.06) | (0.07) | (0.07) | (0.07) | (0.07) | (0.07) |
| Level of WFC in 2019 | -0.02 | -0.02 | 0.01 | 0.01 | 0.01 | 0.01 |
|  | (0.02) | (0.02) | (0.02) | (0.02) | (0.02) | (0.02) |
| Full-time work | -0.00 | 0.00 | 0.01 | 0.01 | 0.01 | 0.01 |
|  | (0.08) | (0.08) | (0.08) | (0.08) | (0.08) | (0.08) |
| Supervisory position | 0.11 | 0.10 | 0.09 | 0.10 | 0.09 | 0.11 |
|  | (0.07) | (0.07) | (0.07) | (0.07) | (0.07) | (0.07) |
| Essential occupation | 0.03 | 0.02 | 0.02 | 0.02 | 0.02 | 0.02 |
|  | (0.07) | (0.07) | (0.07) | (0.07) | (0.07) | (0.07) |
| Tertiary education | -0.11 | -0.09 | -0.10 | -0.10 | -0.10 | -0.10 |
|  | (0.08) | (0.07) | (0.07) | (0.07) | (0.08) | (0.08) |
| Resp. and/or parent(s) born outside Germany | 0.23^**^ | 0.24^**^ | 0.25^**^ | 0.24^**^ | 0.25^**^ | 0.24^**^ |
|  | (0.09) | (0.09) | (0.09) | (0.09) | (0.09) | (0.09) |
| Age | 0.00 | 0.00 | 0.00 | 0.00 | 0.00 | 0.00 |
|  | (0.01) | (0.01) | (0.01) | (0.01) | (0.01) | (0.01) |
| No. of children: 1 | 0.15 | 0.15 | 0.16 | 0.16 | 0.16 | 0.16 |
|  | (0.09) | (0.09) | (0.09) | (0.09) | (0.09) | (0.09) |
| No. of children: 3 or more | -0.21^*^ | -0.21^*^ | -0.21^*^ | -0.21^*^ | -0.21^*^ | -0.21^*^ |
|  | (0.09) | (0.09) | (0.09) | (0.09) | (0.09) | (0.09) |
| At least one child 0–2 years | 0.34^**^ | 0.33^**^ | 0.31^*^ | 0.31^*^ | 0.31^*^ | 0.32^*^ |
|  | (0.12) | (0.12) | (0.12) | (0.12) | (0.12) | (0.12) |
| At least one child 3–5 years | 0.12 | 0.13 | 0.11 | 0.11 | 0.11 | 0.11 |
|  | (0.09) | (0.09) | (0.09) | (0.09) | (0.09) | (0.09) |
| At least one child 6–9 years | 0.19^**^ | 0.20^**^ | 0.18^*^ | 0.18^*^ | 0.19^*^ | 0.19^*^ |
|  | (0.07) | (0.07) | (0.07) | (0.07) | (0.07) | (0.07) |
| At least one child 10–11 years | 0.08 | 0.08 | 0.07 | 0.07 | 0.07 | 0.08 |
|  | (0.09) | (0.09) | (0.08) | (0.08) | (0.08) | (0.08) |
| Constant | -0.06 | -0.03 | -0.01 | -0.00 | -0.03 | -0.12 |
|  | (0.53) | (0.51) | (0.51) | (0.50) | (0.51) | (0.51) |
| *Selection equation of participation in the follow-up study* |  |  |  |  |  |  |
| Age | 0.01^*^ | 0.01^*^ | 0.01^*^ | 0.01^*^ | 0.01^*^ | 0.01^*^ |
|  | (0.00) | (0.00) | (0.00) | (0.00) | (0.00) | (0.00) |
| Primary education | -0.31^*^ | -0.31^*^ | -0.31^*^ | -0.31^*^ | -0.31^*^ | -0.31^*^ |
|  | (0.14) | (0.14) | (0.14) | (0.14) | (0.14) | (0.14) |
| Tertiary education | 0.28^***^ | 0.28^***^ | 0.28^***^ | 0.28^***^ | 0.28^***^ | 0.28^***^ |
|  | (0.08) | (0.08) | (0.08) | (0.08) | (0.08) | (0.08) |
| Respondent born outside Germany | -0.35^***^ | -0.35^***^ | -0.35^***^ | -0.35^***^ | -0.35^***^ | -0.35^***^ |
|  | (0.10) | (0.10) | (0.10) | (0.10) | (0.10) | (0.10) |
| Parent(s) born outside Germany | -0.05 | -0.04 | -0.04 | -0.04 | -0.04 | -0.04 |
|  | (0.12) | (0.12) | (0.12) | (0.12) | (0.12) | (0.12) |
| Manager | -0.15 | -0.15 | -0.15 | -0.15 | -0.15 | -0.15 |
|  | (0.15) | (0.15) | (0.15) | (0.15) | (0.15) | (0.15) |
| Professional | -0.17 | -0.17 | -0.17 | -0.17 | -0.17 | -0.16 |
|  | (0.11) | (0.11) | (0.11) | (0.11) | (0.11) | (0.11) |
| Technician or associate professional | -0.22^*^ | -0.23^*^ | -0.23^*^ | -0.23^*^ | -0.23^*^ | -0.22^*^ |
|  | (0.11) | (0.11) | (0.11) | (0.11) | (0.11) | (0.11) |
| Service or sale worker | -0.21 | -0.22 | -0.22 | -0.22 | -0.22 | -0.22 |
|  | (0.13) | (0.13) | (0.13) | (0.13) | (0.13) | (0.13) |
| Elementary occupation | -0.46^*^ | -0.45^*^ | -0.45^*^ | -0.45^*^ | -0.45^*^ | -0.45^*^ |
|  | (0.22) | (0.22) | (0.22) | (0.22) | (0.22) | (0.22) |
| Other occupation | -0.26^*^ | -0.26^*^ | -0.25^*^ | -0.25^*^ | -0.25^*^ | -0.25^*^ |
|  | (0.12) | (0.12) | (0.12) | (0.12) | (0.12) | (0.12) |
| Supervisory position | 0.12 | 0.12 | 0.12^*^ | 0.12^*^ | 0.12^*^ | 0.12^*^ |
|  | (0.06) | (0.06) | (0.06) | (0.06) | (0.06) | (0.06) |
| No. of children: 1 | -0.18^*^ | -0.18^*^ | -0.18^*^ | -0.18^*^ | -0.18^*^ | -0.18^*^ |
|  | (0.08) | (0.08) | (0.08) | (0.08) | (0.08) | (0.08) |
| No. of children: 3 or more | -0.25^**^ | -0.25^**^ | -0.25^**^ | -0.25^**^ | -0.25^**^ | -0.25^**^ |
|  | (0.08) | (0.08) | (0.08) | (0.08) | (0.08) | (0.08) |
| Financial strain: To some extent | -0.32^**^ | -0.32^**^ | -0.33^**^ | -0.33^**^ | -0.33^**^ | -0.33^**^ |
|  | (0.10) | (0.10) | (0.10) | (0.10) | (0.10) | (0.10) |
| Financial strain: (Very) high | -0.48^***^ | -0.48^***^ | -0.48^***^ | -0.48^***^ | -0.48^***^ | -0.48^***^ |
|  | (0.13) | (0.13) | (0.13) | (0.13) | (0.13) | (0.13) |
| Constant | -1.19^***^ | -1.18^***^ | -1.18^***^ | -1.18^***^ | -1.18^***^ | -1.18^***^ |
|  | (0.22) | (0.22) | (0.22) | (0.22) | (0.22) | (0.22) |
| athrho | -0.33 | -0.36 | -0.39 | -0.39 | -0.40 | -0.38 |
|  | (0.27) | (0.25) | (0.25) | (0.25) | (0.26) | (0.26) |
| *N* (t1) | 3839 | 3839 | 3839 | 3839 | 3839 | 3839 |
| *N* (t2) | 620 | 620 | 620 | 620 | 620 | 620 |

*Notes*: Unstandardized coefficients; standard errors (in parentheses) adjusted for clustering at the level of households [*N* (t1) = 2848; *N* (t2) = 520]; weighted data; ^*^ *p* < 0.05, ^**^ *p* < 0.01, ^***^ *p* < 0.001. WFH = working from home for more than half of the regular working hours during the first lockdown in spring 2020; WFC = work-to-family conflict; Δ = difference score. Reference categories (model): part-time work; primary or intermediate education; respondent and parents born in Germany; no. of children: 2. Reference categories (selection equation): intermediate education; respondent and parents born in Germany; clerical support worker; no. of children: 2; low financial strain.

Table A7: Heckman sample selection models of Δ WFC on WFH during and before the COVID-19 pandemic

|  | Mothers | | | Fathers | | |
| --- | --- | --- | --- | --- | --- | --- |
|  | (1) | (2) | (3) | (4) | (5) | (6) |
| WFH during the pandemic | 0.41 | 0.38 | 0.20 | -0.27 | -0.22 | -0.13 |
|  | (0.22) | (0.21) | (0.24) | (0.24) | (0.22) | (0.24) |
| WFH before the pandemic |  |  | 0.43^*^ |  |  | -0.27 |
|  |  |  | (0.22) |  |  | (0.29) |
| *Control variables* |  |  |  |  |  |  |
| Higher working hours during lockdown |  | 0.21 | 0.16 |  | 0.65^*^ | 0.68^**^ |
|  |  | (0.22) | (0.22) |  | (0.26) | (0.26) |
| Lower working hours during lockdown |  | -0.21 | -0.21 |  | -0.14 | -0.17 |
|  |  | (0.19) | (0.19) |  | (0.25) | (0.26) |
| Full-time work |  | -0.59^*^ | -0.58^*^ |  | -0.24 | -0.25 |
|  |  | (0.24) | (0.24) |  | (0.37) | (0.38) |
| Supervisory position |  | 0.60^**^ | 0.58^**^ |  | -0.08 | -0.08 |
|  |  | (0.23) | (0.22) |  | (0.25) | (0.25) |
| Essential occupation |  | 0.07 | 0.07 |  | -0.15 | -0.15 |
|  |  | (0.19) | (0.19) |  | (0.28) | (0.27) |
| Tertiary education |  | 0.34 | 0.23 |  | -0.20 | -0.19 |
|  |  | (0.25) | (0.25) |  | (0.30) | (0.29) |
| Resp. and/or parent(s) born outside Germany |  | -0.47 | -0.44 |  | -0.06 | -0.04 |
|  |  | (0.32) | (0.32) |  | (0.40) | (0.37) |
| Age |  | 0.08^***^ | 0.08^***^ |  | -0.03 | -0.03 |
|  |  | (0.02) | (0.02) |  | (0.03) | (0.02) |
| No. of children: 1 |  | 0.10 | 0.10 |  | -0.29 | -0.22 |
|  |  | (0.26) | (0.26) |  | (0.41) | (0.39) |
| No. of children: 3 or more |  | -0.31 | -0.32 |  | 0.30 | 0.34 |
|  |  | (0.28) | (0.28) |  | (0.35) | (0.34) |
| At least one child 0–2 years |  | -0.16 | -0.19 |  | -0.13 | -0.08 |
|  |  | (0.37) | (0.37) |  | (0.34) | (0.34) |
| At least one child 3–5 years |  | 0.62^**^ | 0.67^**^ |  | 0.53^*^ | 0.53^*^ |
|  |  | (0.22) | (0.23) |  | (0.26) | (0.26) |
| At least one child 6–9 years |  | 0.34 | 0.32 |  | -0.09 | -0.08 |
|  |  | (0.22) | (0.22) |  | (0.23) | (0.24) |
| At least one child 10–11 years |  | 0.22 | 0.21 |  | -0.04 | -0.01 |
|  |  | (0.24) | (0.23) |  | (0.34) | (0.33) |
| Constant | -0.24 | -6.76^***^ | -6.76^***^ | 1.58^*^ | 3.01 | 3.25^*^ |
|  | (1.04) | (1.18) | (1.20) | (0.74) | (1.77) | (1.63) |
| *Selection equation of participation in the follow-up study* |  |  |  |  |  |  |
| Age | 0.02^*^ | 0.02^**^ | 0.02^**^ | 0.00 | 0.01 | 0.01 |
|  | (0.01) | (0.01) | (0.01) | (0.01) | (0.01) | (0.01) |
| Primary education | -0.40 | -0.41 | -0.40 | -0.29^*^ | -0.28 | -0.29^*^ |
|  | (0.27) | (0.22) | (0.22) | (0.14) | (0.15) | (0.14) |
| Tertiary education | 0.27^**^ | 0.31^***^ | 0.29^**^ | 0.30^**^ | 0.32^**^ | 0.32^**^ |
|  | (0.10) | (0.09) | (0.09) | (0.11) | (0.12) | (0.12) |
| Respondent born outside Germany | -0.25 | -0.26^*^ | -0.26^*^ | -0.46^***^ | -0.45^***^ | -0.45^***^ |
|  | (0.14) | (0.13) | (0.13) | (0.12) | (0.12) | (0.12) |
| Parent(s) born outside Germany | -0.08 | -0.09 | -0.09 | -0.08 | -0.09 | -0.10 |
|  | (0.16) | (0.14) | (0.14) | (0.15) | (0.18) | (0.17) |
| Manager | -0.05 | -0.16 | -0.15 | -0.41 | -0.41^*^ | -0.40 |
|  | (0.23) | (0.18) | (0.18) | (0.21) | (0.21) | (0.21) |
| Professional | -0.02 | -0.13 | -0.11 | -0.49^**^ | -0.51^**^ | -0.50^**^ |
|  | (0.16) | (0.11) | (0.11) | (0.17) | (0.17) | (0.17) |
| Technician or associate professional | -0.06 | -0.11 | -0.12 | -0.64^***^ | -0.65^***^ | -0.64^***^ |
|  | (0.14) | (0.10) | (0.11) | (0.18) | (0.18) | (0.18) |
| Service or sale worker | -0.05 | -0.10 | -0.11 | -0.57^*^ | -0.53^*^ | -0.52^*^ |
|  | (0.16) | (0.13) | (0.13) | (0.23) | (0.25) | (0.24) |
| Elementary occupation | -0.74^*^ | -0.78^**^ | -0.80^**^ | -0.39 | -0.36 | -0.35 |
|  | (0.30) | (0.26) | (0.26) | (0.29) | (0.32) | (0.31) |
| Other occupation | -0.02 | -0.08 | -0.10 | -0.60^***^ | -0.59^***^ | -0.59^***^ |
|  | (0.17) | (0.14) | (0.14) | (0.17) | (0.17) | (0.17) |
| Supervisory position | 0.07 | 0.10 | 0.09 | 0.22^*^ | 0.22^*^ | 0.22^*^ |
|  | (0.10) | (0.09) | (0.09) | (0.09) | (0.09) | (0.09) |
| No. of children: 1 | -0.08 | -0.07 | -0.07 | -0.38^***^ | -0.34^**^ | -0.34^**^ |
|  | (0.10) | (0.09) | (0.09) | (0.11) | (0.12) | (0.12) |
| No. of children: 3 or more | -0.23^*^ | -0.24^*^ | -0.24^*^ | -0.25^*^ | -0.28^**^ | -0.28^**^ |
|  | (0.10) | (0.10) | (0.10) | (0.10) | (0.10) | (0.10) |
| Financial strain: To some extent | -0.37^**^ | -0.33^**^ | -0.33^**^ | -0.14 | -0.16 | -0.15 |
|  | (0.13) | (0.11) | (0.11) | (0.13) | (0.14) | (0.13) |
| Financial strain: (Very) high | -0.53^***^ | -0.39^**^ | -0.39^**^ | -0.43^*^ | -0.43^*^ | -0.43^*^ |
|  | (0.16) | (0.15) | (0.15) | (0.20) | (0.20) | (0.19) |
| Constant | -1.50^***^ | -1.51^***^ | -1.49^***^ | -0.56 | -0.78^*^ | -0.78^*^ |
|  | (0.37) | (0.28) | (0.28) | (0.33) | (0.32) | (0.32) |
| athrho | 0.04 | 1.19^***^ | 1.18^***^ | -0.64^*^ | -0.68 | -0.76 |
|  | (0.43) | (0.25) | (0.26) | (0.30) | (0.47) | (0.42) |
| *N* (t1) | 2053 | 2053 | 2053 | 1786 | 1786 | 1786 |
| *N* (t2) | 337 | 337 | 337 | 283 | 283 | 283 |

*Notes*: Unstandardized coefficients; robust standard errors (in parentheses); weighted data; ^*^ *p* < 0.05, ^**^ *p* < 0.01, ^***^ *p* < 0.001. WFC = work-to-family conflict; Δ = difference score; WFH during the pandemic = working from home for more than half of the regular working hours during the first lockdown in spring 2020 (1 = yes); WFH before the pandemic = use of work from home in 2019 (1 = yes). Reference categories (model): part-time work; primary or intermediate education; respondent and parents born in Germany; no. of children: 2. Reference categories (selection equation): intermediate education; respondent and parents born in Germany; clerical support worker; no. of children: 2; low financial strain.

Table A8: Heckman sample selection models of Δ responsive parenting and Δ harsh parenting among partnered mothers

|  | Responsive | | |  | Harsh |  |
| --- | --- | --- | --- | --- | --- | --- |
|  | (1) | (2) | (3) | (4) | (5) | (6) |
| WFH | 0.14 | 0.13 | 0.13 | -0.17 | -0.16 | -0.16 |
|  | (0.13) | (0.12) | (0.12) | (0.09) | (0.09) | (0.09) |
| Δ WFC |  | -0.07 | -0.16^**^ |  | 0.05 | 0.06 |
|  |  | (0.04) | (0.05) |  | (0.03) | (0.04) |
| WFH # Δ WFC |  |  | 0.23^***^ |  |  | -0.02 |
|  |  |  | (0.07) |  |  | (0.05) |
| *Control variables* |  |  |  |  |  |  |
| Higher working hours during lockdown | -0.10 | -0.07 | -0.07 | 0.00 | -0.02 | -0.02 |
|  | (0.12) | (0.13) | (0.12) | (0.10) | (0.10) | (0.10) |
| Lower working hours during lockdown | -0.03 | -0.01 | 0.00 | 0.04 | 0.02 | 0.02 |
|  | (0.12) | (0.12) | (0.12) | (0.09) | (0.09) | (0.09) |
| Use of WFH in 2019 | 0.02 | 0.04 | 0.03 | 0.08 | 0.07 | 0.07 |
|  | (0.14) | (0.14) | (0.14) | (0.09) | (0.09) | (0.09) |
| Level of WFC in 2019 | -0.02 | -0.07 | -0.06 | 0.01 | 0.04 | 0.04 |
|  | (0.04) | (0.05) | (0.05) | (0.03) | (0.03) | (0.03) |
| Full-time work | -0.03 | -0.05 | -0.05 | 0.06 | 0.07 | 0.08 |
|  | (0.12) | (0.13) | (0.12) | (0.11) | (0.11) | (0.11) |
| Supervisory position | -0.17 | -0.15 | -0.22 | 0.11 | 0.10 | 0.11 |
|  | (0.13) | (0.13) | (0.13) | (0.10) | (0.10) | (0.10) |
| Essential occupation | 0.25^*^ | 0.26^*^ | 0.28^*^ | 0.04 | 0.03 | 0.03 |
|  | (0.13) | (0.13) | (0.12) | (0.10) | (0.09) | (0.09) |
| Tertiary education | -0.46^**^ | -0.44^*^ | -0.46^**^ | -0.31^**^ | -0.33^**^ | -0.33^**^ |
|  | (0.18) | (0.18) | (0.17) | (0.11) | (0.11) | (0.11) |
| Resp. and/or parent(s) born outside Germany | 0.17 | 0.15 | 0.21 | 0.30^*^ | 0.31^*^ | 0.31^*^ |
|  | (0.17) | (0.17) | (0.17) | (0.14) | (0.14) | (0.14) |
| Age | -0.01 | -0.01 | -0.01 | -0.01 | -0.01 | -0.01 |
|  | (0.01) | (0.01) | (0.01) | (0.01) | (0.01) | (0.01) |
| No. of children: 1 | -0.04 | -0.02 | -0.03 | 0.08 | 0.07 | 0.07 |
|  | (0.15) | (0.15) | (0.15) | (0.11) | (0.11) | (0.11) |
| No. of children: 3 or more | 0.49^**^ | 0.48^**^ | 0.49^**^ | -0.06 | -0.05 | -0.05 |
|  | (0.17) | (0.17) | (0.16) | (0.11) | (0.11) | (0.11) |
| At least one child 0–2 years | -0.02 | 0.02 | -0.04 | 0.13 | 0.10 | 0.10 |
|  | (0.19) | (0.19) | (0.20) | (0.15) | (0.15) | (0.15) |
| At least one child 3–5 years | -0.03 | 0.01 | -0.01 | 0.11 | 0.08 | 0.08 |
|  | (0.14) | (0.14) | (0.14) | (0.13) | (0.13) | (0.13) |
| At least one child 6–9 years | -0.02 | 0.03 | 0.06 | 0.25^**^ | 0.21^*^ | 0.21^*^ |
|  | (0.12) | (0.12) | (0.11) | (0.09) | (0.10) | (0.09) |
| At least one child 10–11 years | -0.34^**^ | -0.30^*^ | -0.34^**^ | -0.07 | -0.10 | -0.10 |
|  | (0.13) | (0.13) | (0.13) | (0.10) | (0.10) | (0.10) |
| Constant | 1.73^*^ | 1.57^*^ | 1.69^*^ | 1.27^*^ | 1.41^**^ | 1.41^**^ |
|  | (0.76) | (0.77) | (0.72) | (0.52) | (0.53) | (0.53) |
| *Selection equation of participation in the follow-up study* |  |  |  |  |  |  |
| Age | 0.02^**^ | 0.02^**^ | 0.02^**^ | 0.02^**^ | 0.02^**^ | 0.02^**^ |
|  | (0.01) | (0.01) | (0.01) | (0.01) | (0.01) | (0.01) |
| Primary education | -0.41 | -0.41 | -0.41 | -0.40 | -0.40 | -0.40 |
|  | (0.26) | (0.26) | (0.25) | (0.24) | (0.23) | (0.23) |
| Tertiary education | 0.23^*^ | 0.23^*^ | 0.23^*^ | 0.25^*^ | 0.25^*^ | 0.25^*^ |
|  | (0.10) | (0.10) | (0.10) | (0.10) | (0.10) | (0.10) |
| Respondent born outside Germany | -0.21 | -0.21 | -0.21 | -0.21 | -0.21 | -0.21 |
|  | (0.14) | (0.14) | (0.14) | (0.14) | (0.14) | (0.14) |
| Parent(s) born outside Germany | 0.06 | 0.06 | 0.07 | 0.09 | 0.09 | 0.09 |
|  | (0.16) | (0.16) | (0.15) | (0.18) | (0.18) | (0.18) |
| Manager | 0.00 | 0.02 | -0.02 | -0.16 | -0.19 | -0.19 |
|  | (0.21) | (0.22) | (0.21) | (0.22) | (0.22) | (0.22) |
| Professional | -0.01 | -0.01 | -0.01 | -0.03 | -0.04 | -0.04 |
|  | (0.12) | (0.12) | (0.12) | (0.13) | (0.13) | (0.13) |
| Technician or associate professional | -0.09 | -0.09 | -0.09 | -0.14 | -0.15 | -0.14 |
|  | (0.12) | (0.12) | (0.11) | (0.13) | (0.12) | (0.12) |
| Service or sale worker | -0.27 | -0.26 | -0.27 | -0.19 | -0.19 | -0.19 |
|  | (0.15) | (0.15) | (0.15) | (0.16) | (0.16) | (0.16) |
| Elementary occupation | -0.70^**^ | -0.68^**^ | -0.65^**^ | -0.53 | -0.53 | -0.54 |
|  | (0.25) | (0.25) | (0.25) | (0.29) | (0.29) | (0.29) |
| Other occupation | -0.08 | -0.09 | -0.08 | 0.01 | 0.01 | 0.01 |
|  | (0.17) | (0.17) | (0.17) | (0.17) | (0.17) | (0.17) |
| Supervisory position | -0.01 | -0.01 | -0.01 | 0.01 | 0.02 | 0.02 |
|  | (0.09) | (0.09) | (0.09) | (0.10) | (0.10) | (0.10) |
| No. of children: 1 | -0.07 | -0.07 | -0.07 | -0.05 | -0.05 | -0.05 |
|  | (0.10) | (0.10) | (0.10) | (0.10) | (0.10) | (0.10) |
| No. of children: 3 or more | -0.27^**^ | -0.27^**^ | -0.28^**^ | -0.26^**^ | -0.26^**^ | -0.26^**^ |
|  | (0.10) | (0.10) | (0.10) | (0.10) | (0.10) | (0.10) |
| Financial strain: To some extent | -0.29^*^ | -0.29^*^ | -0.28^*^ | -0.44^***^ | -0.45^***^ | -0.45^***^ |
|  | (0.14) | (0.15) | (0.14) | (0.13) | (0.13) | (0.13) |
| Financial strain: (Very) high | -0.46^**^ | -0.47^**^ | -0.44^*^ | -0.50^*^ | -0.49^*^ | -0.50^*^ |
|  | (0.16) | (0.17) | (0.17) | (0.19) | (0.19) | (0.19) |
| Constant | -1.59^***^ | -1.59^***^ | -1.59^***^ | -1.54^***^ | -1.53^***^ | -1.53^***^ |
|  | (0.31) | (0.31) | (0.31) | (0.31) | (0.31) | (0.31) |
| athrho | -1.07^***^ | -1.03^***^ | -1.09^***^ | -0.91^**^ | -0.97^***^ | -0.97^***^ |
|  | (0.28) | (0.30) | (0.27) | (0.29) | (0.29) | (0.29) |
| *N* (t1) | 1768 | 1768 | 1768 | 1768 | 1768 | 1768 |
| *N* (t2) | 301 | 301 | 301 | 301 | 301 | 301 |

*Notes*: Unstandardized coefficients; robust standard errors (in parentheses); weighted data; ^*^ *p* < 0.05, ^**^ *p* < 0.01, ^***^ *p* < 0.001. WFH = working from home for more than half of the regular working hours during the first lockdown in spring 2020; WFC = work-to-family conflict; Δ = difference score. Reference categories (model): part-time work; primary or intermediate education; respondent and parents born in Germany; no. of children: 2. Reference categories (selection equation): intermediate education; respondent and parents born in Germany; clerical support worker; no. of children: 2; low financial strain.

Table A9: Heckman sample selection models of gender differences in Δ responsive parenting among partnered parents

|  | (1) | (2) | (3) | (4) | (5) | (6) |
| --- | --- | --- | --- | --- | --- | --- |
| Female | -0.15 | -0.14 | -0.15 | -0.17 | -0.16 | -0.20 |
|  | (0.11) | (0.11) | (0.11) | (0.11) | (0.14) | (0.14) |
| WFH |  | 0.16 | 0.15 | 0.16 | 0.14 | 0.13 |
|  |  | (0.09) | (0.09) | (0.09) | (0.12) | (0.13) |
| Δ WFC |  |  | -0.08^*^ | -0.12^**^ | -0.08^*^ | -0.09 |
|  |  |  | (0.03) | (0.04) | (0.03) | (0.06) |
| WFH # Δ WFC |  |  |  | 0.10^*^ |  | 0.00 |
|  |  |  |  | (0.05) |  | (0.07) |
| WFH # Female |  |  |  |  | 0.03 | 0.03 |
|  |  |  |  |  | (0.17) | (0.16) |
| Δ WFC # Female |  |  |  |  |  | -0.06 |
|  |  |  |  |  |  | (0.07) |
| WFH # Δ WFC # Female |  |  |  |  |  | 0.21^*^ |
|  |  |  |  |  |  | (0.10) |
| *Control variables* |  |  |  |  |  |  |
| Higher working hours during lockdown | -0.01 | -0.02 | 0.03 | 0.02 | 0.02 | 0.03 |
|  | (0.10) | (0.10) | (0.10) | (0.10) | (0.10) | (0.10) |
| Lower working hours during lockdown | 0.10 | 0.11 | 0.11 | 0.11 | 0.11 | 0.11 |
|  | (0.10) | (0.10) | (0.10) | (0.10) | (0.10) | (0.10) |
| Use of WFH in 2019 | 0.02 | -0.04 | -0.04 | -0.03 | -0.04 | -0.04 |
|  | (0.09) | (0.09) | (0.09) | (0.09) | (0.09) | (0.09) |
| Level of WFC in 2019 | -0.00 | -0.00 | -0.05 | -0.05 | -0.05 | -0.05 |
|  | (0.03) | (0.03) | (0.04) | (0.04) | (0.04) | (0.04) |
| Full-time work | -0.01 | -0.00 | -0.02 | -0.02 | -0.02 | -0.02 |
|  | (0.11) | (0.10) | (0.10) | (0.10) | (0.10) | (0.11) |
| Supervisory position | -0.23^*^ | -0.22^*^ | -0.20^*^ | -0.21^*^ | -0.20^*^ | -0.24^*^ |
|  | (0.10) | (0.10) | (0.10) | (0.10) | (0.10) | (0.10) |
| Essential occupation | 0.28^**^ | 0.28^**^ | 0.29^***^ | 0.29^***^ | 0.29^***^ | 0.30^***^ |
|  | (0.09) | (0.09) | (0.09) | (0.09) | (0.09) | (0.09) |
| Tertiary education | -0.27^*^ | -0.29^*^ | -0.28^*^ | -0.30^**^ | -0.27^*^ | -0.29^*^ |
|  | (0.11) | (0.11) | (0.11) | (0.11) | (0.11) | (0.12) |
| Resp. and/or parent(s) born outside Germany | 0.23^*^ | 0.21^*^ | 0.20 | 0.21^*^ | 0.20 | 0.23^*^ |
|  | (0.11) | (0.11) | (0.11) | (0.11) | (0.11) | (0.11) |
| Age | 0.02 | 0.02 | 0.02 | 0.02 | 0.02 | 0.01 |
|  | (0.01) | (0.01) | (0.01) | (0.01) | (0.01) | (0.01) |
| No. of children: 1 | -0.05 | -0.07 | -0.07 | -0.05 | -0.07 | -0.06 |
|  | (0.12) | (0.12) | (0.11) | (0.11) | (0.11) | (0.12) |
| No. of children: 3 or more | 0.38^**^ | 0.38^**^ | 0.37^**^ | 0.38^**^ | 0.37^**^ | 0.39^**^ |
|  | (0.13) | (0.13) | (0.13) | (0.13) | (0.13) | (0.13) |
| At least one child 0–2 years | -0.03 | -0.01 | 0.02 | 0.02 | 0.02 | -0.00 |
|  | (0.13) | (0.13) | (0.13) | (0.13) | (0.13) | (0.13) |
| At least one child 3–5 years | 0.06 | 0.05 | 0.08 | 0.08 | 0.08 | 0.07 |
|  | (0.11) | (0.11) | (0.11) | (0.11) | (0.11) | (0.11) |
| At least one child 6–9 years | -0.00 | -0.01 | 0.01 | 0.03 | 0.01 | 0.02 |
|  | (0.09) | (0.09) | (0.09) | (0.09) | (0.09) | (0.09) |
| At least one child 10–11 years | -0.22^*^ | -0.24^*^ | -0.20^*^ | -0.20 | -0.20^*^ | -0.22^*^ |
|  | (0.10) | (0.10) | (0.10) | (0.10) | (0.10) | (0.11) |
| Constant | 0.07 | -0.08 | -0.05 | -0.01 | -0.04 | 0.15 |
|  | (0.66) | (0.68) | (0.68) | (0.69) | (0.68) | (0.73) |
| *Selection equation of participation in the follow-up study* |  |  |  |  |  |  |
| Age | 0.01^**^ | 0.01^**^ | 0.01^**^ | 0.01^**^ | 0.01^**^ | 0.01^**^ |
|  | (0.00) | (0.00) | (0.00) | (0.00) | (0.00) | (0.00) |
| Primary education | -0.33^*^ | -0.33^*^ | -0.33^*^ | -0.33^*^ | -0.33^*^ | -0.33^*^ |
|  | (0.15) | (0.15) | (0.15) | (0.15) | (0.15) | (0.15) |
| Tertiary education | 0.28^***^ | 0.28^***^ | 0.28^***^ | 0.28^***^ | 0.28^***^ | 0.28^***^ |
|  | (0.08) | (0.08) | (0.08) | (0.08) | (0.08) | (0.08) |
| Respondent born outside Germany | -0.31^**^ | -0.31^**^ | -0.31^**^ | -0.31^**^ | -0.31^**^ | -0.31^**^ |
|  | (0.11) | (0.11) | (0.11) | (0.11) | (0.11) | (0.11) |
| Parent(s) born outside Germany | 0.04 | 0.04 | 0.04 | 0.04 | 0.04 | 0.04 |
|  | (0.12) | (0.12) | (0.12) | (0.12) | (0.12) | (0.12) |
| Manager | -0.16 | -0.16 | -0.16 | -0.16 | -0.15 | -0.16 |
|  | (0.15) | (0.15) | (0.15) | (0.15) | (0.15) | (0.15) |
| Professional | -0.20 | -0.20 | -0.20 | -0.20 | -0.20 | -0.20 |
|  | (0.12) | (0.12) | (0.12) | (0.12) | (0.12) | (0.12) |
| Technician or associate professional | -0.30^**^ | -0.29^*^ | -0.29^*^ | -0.29^*^ | -0.29^*^ | -0.29^*^ |
|  | (0.11) | (0.12) | (0.12) | (0.12) | (0.12) | (0.12) |
| Service or sale worker | -0.32^*^ | -0.31^*^ | -0.31^*^ | -0.31^*^ | -0.31^*^ | -0.31^*^ |
|  | (0.14) | (0.14) | (0.14) | (0.14) | (0.14) | (0.14) |
| Elementary occupation | -0.50^*^ | -0.50^*^ | -0.49^*^ | -0.48^*^ | -0.49^*^ | -0.48^*^ |
|  | (0.21) | (0.21) | (0.21) | (0.21) | (0.21) | (0.21) |
| Other occupation | -0.30^*^ | -0.30^*^ | -0.30^*^ | -0.30^*^ | -0.30^*^ | -0.30^*^ |
|  | (0.12) | (0.12) | (0.12) | (0.12) | (0.12) | (0.12) |
| Supervisory position | 0.07 | 0.07 | 0.07 | 0.07 | 0.07 | 0.07 |
|  | (0.06) | (0.06) | (0.06) | (0.06) | (0.06) | (0.06) |
| No. of children: 1 | -0.17 | -0.17 | -0.17 | -0.17 | -0.17 | -0.17 |
|  | (0.09) | (0.09) | (0.09) | (0.09) | (0.09) | (0.09) |
| No. of children: 3 or more | -0.29^***^ | -0.29^***^ | -0.29^***^ | -0.29^***^ | -0.29^***^ | -0.29^***^ |
|  | (0.08) | (0.08) | (0.08) | (0.08) | (0.08) | (0.08) |
| Financial strain: To some extent | -0.27^*^ | -0.28^*^ | -0.28^*^ | -0.27^*^ | -0.28^*^ | -0.27^*^ |
|  | (0.12) | (0.12) | (0.12) | (0.12) | (0.12) | (0.13) |
| Financial strain: (Very) high | -0.43^**^ | -0.43^**^ | -0.44^**^ | -0.43^**^ | -0.44^**^ | -0.43^**^ |
|  | (0.16) | (0.16) | (0.16) | (0.16) | (0.16) | (0.15) |
| Constant | -1.19^***^ | -1.19^***^ | -1.19^***^ | -1.19^***^ | -1.19^***^ | -1.19^***^ |
|  | (0.23) | (0.23) | (0.23) | (0.23) | (0.23) | (0.23) |
| athrho | -0.56^*^ | -0.50 | -0.47 | -0.50 | -0.47 | -0.54 |
|  | (0.25) | (0.26) | (0.26) | (0.27) | (0.26) | (0.30) |
| *N* (t1) | 3511 | 3511 | 3511 | 3511 | 3511 | 3511 |
| *N* (t2) | 570 | 570 | 570 | 570 | 570 | 570 |

*Notes*: Unstandardized coefficients; standard errors (in parentheses) adjusted for clustering at the level of households [*N* (t1) = 2530; *N* (t2) = 471]; weighted data; ^*^ *p* < 0.05, ^**^ *p* < 0.01, ^***^ *p* < 0.001. WFH = working from home for more than half of the regular working hours during the first lockdown in spring 2020; WFC = work-to-family conflict; Δ = difference score. Reference categories (model): part-time work; primary or intermediate education; respondent and parents born in Germany; no. of children: 2. Reference categories (selection equation): intermediate education; respondent and parents born in Germany; clerical support worker; no. of children: 2; low financial strain.

Table A10: Heckman sample selection models of gender differences in Δ harsh parenting among partnered parents

|  | (1) | (2) | (3) | (4) | (5) | (6) |
| --- | --- | --- | --- | --- | --- | --- |
| Female | 0.22^*^ | 0.22^*^ | 0.22^*^ | 0.22^*^ | 0.26^*^ | 0.26^*^ |
|  | (0.09) | (0.09) | (0.09) | (0.09) | (0.11) | (0.11) |
| WFH |  | -0.08 | -0.08 | -0.08 | -0.04 | -0.03 |
|  |  | (0.08) | (0.08) | (0.08) | (0.11) | (0.11) |
| Δ WFC |  |  | 0.05^*^ | 0.05 | 0.05^*^ | 0.05 |
|  |  |  | (0.02) | (0.03) | (0.02) | (0.05) |
| WFH # Δ WFC |  |  |  | -0.00 |  | 0.03 |
|  |  |  |  | (0.04) |  | (0.06) |
| WFH # Female |  |  |  |  | -0.07 | -0.07 |
|  |  |  |  |  | (0.13) | (0.13) |
| Δ WFC # Female |  |  |  |  |  | -0.01 |
|  |  |  |  |  |  | (0.05) |
| WFH # Δ WFC # Female |  |  |  |  |  | -0.06 |
|  |  |  |  |  |  | (0.07) |
| *Control variables* |  |  |  |  |  |  |
| Higher working hours during lockdown | 0.04 | 0.04 | 0.01 | 0.01 | 0.01 | 0.01 |
|  | (0.08) | (0.08) | (0.08) | (0.08) | (0.08) | (0.08) |
| Lower working hours during lockdown | 0.14 | 0.14 | 0.14 | 0.14 | 0.14 | 0.14 |
|  | (0.08) | (0.08) | (0.08) | (0.08) | (0.08) | (0.08) |
| Use of WFH in 2019 | -0.07 | -0.04 | -0.05 | -0.05 | -0.05 | -0.04 |
|  | (0.07) | (0.07) | (0.07) | (0.07) | (0.07) | (0.07) |
| Level of WFC in 2019 | -0.02 | -0.02 | 0.01 | 0.01 | 0.01 | 0.01 |
|  | (0.02) | (0.02) | (0.03) | (0.03) | (0.03) | (0.03) |
| Full-time work | 0.03 | 0.03 | 0.04 | 0.04 | 0.04 | 0.04 |
|  | (0.08) | (0.08) | (0.08) | (0.08) | (0.08) | (0.08) |
| Supervisory position | 0.12 | 0.12 | 0.11 | 0.11 | 0.11 | 0.12 |
|  | (0.07) | (0.07) | (0.07) | (0.07) | (0.07) | (0.07) |
| Essential occupation | 0.06 | 0.06 | 0.06 | 0.06 | 0.06 | 0.05 |
|  | (0.08) | (0.08) | (0.08) | (0.08) | (0.08) | (0.08) |
| Tertiary education | -0.13 | -0.12 | -0.13 | -0.13 | -0.13 | -0.13 |
|  | (0.08) | (0.08) | (0.08) | (0.08) | (0.08) | (0.08) |
| Resp. and/or parent(s) born outside Germany | 0.23^**^ | 0.24^**^ | 0.25^**^ | 0.25^**^ | 0.25^**^ | 0.25^**^ |
|  | (0.08) | (0.08) | (0.08) | (0.08) | (0.08) | (0.09) |
| Age | 0.00 | 0.00 | 0.00 | 0.00 | 0.00 | 0.00 |
|  | (0.01) | (0.01) | (0.01) | (0.01) | (0.01) | (0.01) |
| No. of children: 1 | 0.15 | 0.16 | 0.16 | 0.16 | 0.16 | 0.16 |
|  | (0.10) | (0.10) | (0.10) | (0.10) | (0.10) | (0.10) |
| No. of children: 3 or more | -0.21^*^ | -0.21^*^ | -0.21^*^ | -0.21^*^ | -0.21^*^ | -0.21^*^ |
|  | (0.10) | (0.10) | (0.10) | (0.10) | (0.10) | (0.10) |
| At least one child 0–2 years | 0.29^*^ | 0.28^*^ | 0.26^*^ | 0.26^*^ | 0.26^*^ | 0.27^*^ |
|  | (0.13) | (0.13) | (0.13) | (0.13) | (0.13) | (0.13) |
| At least one child 3–5 years | 0.12 | 0.13 | 0.11 | 0.11 | 0.11 | 0.11 |
|  | (0.09) | (0.09) | (0.09) | (0.09) | (0.09) | (0.09) |
| At least one child 6–9 years | 0.17^*^ | 0.18^*^ | 0.16^*^ | 0.16^*^ | 0.17^*^ | 0.17^*^ |
|  | (0.08) | (0.08) | (0.08) | (0.08) | (0.08) | (0.08) |
| At least one child 10–11 years | 0.07 | 0.08 | 0.06 | 0.06 | 0.06 | 0.07 |
|  | (0.09) | (0.09) | (0.09) | (0.09) | (0.09) | (0.09) |
| Constant | 0.02 | 0.07 | 0.06 | 0.06 | 0.04 | -0.02 |
|  | (0.56) | (0.54) | (0.54) | (0.54) | (0.54) | (0.56) |
| *Selection equation of participation in the follow-up study* |  |  |  |  |  |  |
| Age | 0.01^**^ | 0.01^**^ | 0.01^**^ | 0.01^**^ | 0.01^**^ | 0.01^**^ |
|  | (0.00) | (0.00) | (0.00) | (0.00) | (0.00) | (0.00) |
| Primary education | -0.33^*^ | -0.33^*^ | -0.33^*^ | -0.33^*^ | -0.33^*^ | -0.33^*^ |
|  | (0.15) | (0.15) | (0.15) | (0.15) | (0.15) | (0.15) |
| Tertiary education | 0.28^***^ | 0.28^***^ | 0.28^***^ | 0.28^***^ | 0.28^***^ | 0.28^***^ |
|  | (0.08) | (0.08) | (0.08) | (0.08) | (0.08) | (0.08) |
| Respondent born outside Germany | -0.30^**^ | -0.31^**^ | -0.31^**^ | -0.31^**^ | -0.31^**^ | -0.31^**^ |
|  | (0.11) | (0.11) | (0.11) | (0.11) | (0.11) | (0.11) |
| Parent(s) born outside Germany | 0.03 | 0.03 | 0.04 | 0.04 | 0.04 | 0.04 |
|  | (0.13) | (0.13) | (0.13) | (0.13) | (0.13) | (0.13) |
| Manager | -0.16 | -0.16 | -0.16 | -0.16 | -0.16 | -0.16 |
|  | (0.16) | (0.16) | (0.16) | (0.16) | (0.16) | (0.16) |
| Professional | -0.20 | -0.20 | -0.20 | -0.20 | -0.20 | -0.20 |
|  | (0.12) | (0.12) | (0.12) | (0.12) | (0.12) | (0.12) |
| Technician or associate professional | -0.29^*^ | -0.29^*^ | -0.29^*^ | -0.29^*^ | -0.29^*^ | -0.29^*^ |
|  | (0.12) | (0.12) | (0.12) | (0.12) | (0.12) | (0.12) |
| Service or sale worker | -0.29^*^ | -0.29^*^ | -0.29^*^ | -0.29^*^ | -0.30^*^ | -0.29^*^ |
|  | (0.14) | (0.14) | (0.14) | (0.14) | (0.14) | (0.14) |
| Elementary occupation | -0.43 | -0.42 | -0.42 | -0.42 | -0.42 | -0.42 |
|  | (0.23) | (0.23) | (0.23) | (0.23) | (0.23) | (0.23) |
| Other occupation | -0.29^*^ | -0.30^*^ | -0.29^*^ | -0.29^*^ | -0.29^*^ | -0.29^*^ |
|  | (0.13) | (0.13) | (0.13) | (0.13) | (0.13) | (0.13) |
| Supervisory position | 0.07 | 0.07 | 0.07 | 0.07 | 0.07 | 0.07 |
|  | (0.06) | (0.06) | (0.06) | (0.06) | (0.06) | (0.06) |
| No. of children: 1 | -0.17 | -0.17 | -0.17 | -0.17 | -0.17 | -0.17 |
|  | (0.09) | (0.09) | (0.09) | (0.09) | (0.09) | (0.09) |
| No. of children: 3 or more | -0.29^***^ | -0.29^***^ | -0.29^***^ | -0.29^***^ | -0.29^***^ | -0.29^***^ |
|  | (0.08) | (0.08) | (0.08) | (0.08) | (0.08) | (0.08) |
| Financial strain: To some extent | -0.33^**^ | -0.33^**^ | -0.33^**^ | -0.33^**^ | -0.33^**^ | -0.33^**^ |
|  | (0.11) | (0.11) | (0.11) | (0.11) | (0.11) | (0.11) |
| Financial strain: (Very) high | -0.43^**^ | -0.43^**^ | -0.43^**^ | -0.43^**^ | -0.43^**^ | -0.43^**^ |
|  | (0.16) | (0.16) | (0.16) | (0.16) | (0.16) | (0.16) |
| Constant | -1.18^***^ | -1.18^***^ | -1.18^***^ | -1.18^***^ | -1.18^***^ | -1.18^***^ |
|  | (0.23) | (0.23) | (0.23) | (0.23) | (0.23) | (0.23) |
| athrho | -0.29 | -0.32 | -0.35 | -0.35 | -0.35 | -0.34 |
|  | (0.27) | (0.25) | (0.25) | (0.25) | (0.26) | (0.27) |
| *N* (t1) | 3511 | 3511 | 3511 | 3511 | 3511 | 3511 |
| *N* (t2) | 570 | 570 | 570 | 570 | 570 | 570 |

*Notes*: Unstandardized coefficients; standard errors (in parentheses) adjusted for clustering at the level of households [*N* (t1) = 2530; *N* (t2) = 471]; weighted data; ^*^ *p* < 0.05, ^**^ *p* < 0.01, ^***^ *p* < 0.001. WFH = working from home for more than half of the regular working hours during the first lockdown in spring 2020; WFC = work-to-family conflict; Δ = difference score. Reference categories (model): part-time work; primary or intermediate education; respondent and parents born in Germany; no. of children: 2. Reference categories (selection equation): intermediate education; respondent and parents born in Germany; clerical support worker; no. of children: 2; low financial strain.
